# Visual response properties of neurons in the superficial layers of the superior colliculus of awake mouse

**DOI:** 10.1113/JP276964

**Published:** 2018-11-10

**Authors:** Gioia De Franceschi, Samuel G. Solomon

**Affiliations:** ^1^ Institute of Behavioural Neuroscience, Department of Experimental Psychology University College London London UK

**Keywords:** vision, receptive field, functional properties, anaesthesia, tectum

## Abstract

**Key points:**

- In rodents, including mice, the superior colliculus is the major target of the retina, but its visual response is not well characterized.
- In the present study, extracellular recordings from single nerve cells in the superficial layers of the superior colliculus were made in awake, head‐restrained mice, and their responses to visual stimuli were measured.
- It was found that these neurons show brisk, highly sensitive and short latency visual responses, a preference for black over white stimuli, and diverse responses to moving patterns.
- At least five broad classes can be defined by variation in functional properties among units.
- The results of the present study demonstrate that eye movements have a measurable impact on visual responses in awake animals and show how they may be mitigated in analyses.

**Abstract:**

The mouse is an increasingly important animal model of visual function in health and disease. In mice, most retinal signals are routed through the superficial layers of the midbrain superior colliculus, and it is well established that much of the visual behaviour of mice relies on activity in the superior colliculus. The functional organization of visual signals in the mouse superior colliculus is, however, not well established in awake animals. We therefore made extracellular recordings from the superficial layers of the superior colliculus in awake mice, while the animals were viewing visual stimuli including flashed spots and drifting gratings. We find that neurons in the superficial layers of the superior colliculus of awake mouse generally show short latency, brisk responses. Receptive fields are usually ‘ON–OFF’ with a preference for black stimuli, and are weakly non‐linear in response to gratings and other forms of luminance modulation. Population responses to drifting gratings are highly contrast sensitive, with a robust response to spatial frequencies above 0.3 cycles degree^−1^ and temporal frequencies above 15 Hz. The receptive fields are also often speed‐tuned or direction‐selective. Analysis of the response across multiple stimulus dimensions reveals at least five functionally distinct groups of units. We also find that eye movements affect measurements of receptive field properties in awake animals, and show how these may be mitigated in analyses. Qualitatively similar responses were obtained in urethane‐anaesthetized animals, although receptive fields in awake animals had higher contrast sensitivity, shorter visual latency and a stronger response to high temporal frequencies.

## Key points

- In rodents, including mice, the superior colliculus is the major target of the retina, but its visual response is not well characterized.
- In the present study, extracellular recordings from single nerve cells in the superficial layers of the superior colliculus were made in awake, head‐restrained mice, and their responses to visual stimuli were measured.
- It was found that these neurons show brisk, highly sensitive and short latency visual responses, a preference for black over white stimuli, and diverse responses to moving patterns.
- At least five broad classes can be defined by variation in functional properties among units.
- The results of the present study demonstrate that eye movements have a measurable impact on visual responses in awake animals and show how they may be mitigated in analyses.

## Introduction

Visual processing in the superior colliculus (SC) is important for the analysis of potentially important objects, and helps orient simple behaviours towards or away from them. In rodents, including mice, the SC is the primary target of the retina, with at least 85% of retinal ganglion cell output sent to the superficial layers of the SC (Ellis *et al*. 2016). Yet, although the SC is the largest visual area in the mouse brain, and a prominent model in developmental work (Huberman *et al*. 2008 *b*; Cang & Feldheim, 2013), less is known about the functional properties of its neurons compared to those of the dorsal lateral geniculate nucleus of the thalamus (dLGN, the target of ∼30% of retinal output) (Piscopo *et al*. 2013; Durand *et al*. 2016; Tang *et al*. 2016) and primary visual cortex (V1) (Niell & Stryker, 2008; Andermann *et al*. 2011; Vaiceliunaite *et al*. 2013; Durand *et al*. 2016).

Previous functional studies of the superficial layers of mouse SC (sSC) have shown that most neurons have ‘ON–OFF’ receptive fields (i.e. respond to both black and white stimuli) and many are sensitive to the orientation of a pattern or its direction of movement (Drager & Hubel, 1975; Wang *et al*. 2010; Gale & Murphy, 2014; Ahmadlou & Heimel, 2015; Feinberg & Meister, 2015; Inayat *et al*. 2015; Ito *et al*. 2017; Shi *et al*. 2017). The visual responses of neurons in the sSC are driven by retinal inputs but are modulated by cortical inputs (Zhao *et al*. 2014; Ahmadlou *et al*. 2017), are reduced under anaesthesia (Zhao *et al*. 2014) and can depend on behavioural state including locomotion (Ito *et al*. 2017). These effects of behaviour and anaesthesia are similar to those observed in the LGN and V1 of mouse (Vaiceliunaite *et al*. 2013; Erisken *et al*. 2014; Durand *et al*. 2016). Yet, although activity of neurons in the sSC is clearly important for mouse vision, basic general knowledge of visual properties in the sSC of awake mice is lacking. For example, we do not know the contrast sensitivity of these neurons or their spatiotemporal resolution. Nor do we know whether neurons in the sSC of awake mice can be grouped into distinct functional channels similar to those shown for neurons in the LGN and V1 of mouse (Gao *et al*. 2010; Piscopo *et al*. 2013).

The present study aimed to provide basic knowledge of the visual functional properties of neurons in the superficial layers of the SC in awake mouse, how these functional properties are correlated in individual neurons, and thus how these properties may constrain behaviour. We therefore made systematic measurements of receptive field properties using extracellular recordings of single‐units in awake mice.

## Methods

### Ethical approval

All animal care and experimental procedures were conducted in accordance with the UK Animals Scientific Procedures Act (1986) and with the ethical policy under which *The Journal of Physiology* operates. Experiments were performed at University College London under personal and project licenses (70/8637) released by the UK Home Office following appropriate institutional ethics review.

### General

Adult C57BL/6 male mice (aged 8–12 weeks at the start of experiments, weighing 20–35 g) were obtained from Charles River Laboratories (Margate, UK). Animals were housed with food and water *ad libitum*, under a 12:12 h light/dark cycle. Measurements were obtained during the dark phase. On the day of surgery, anaesthesia was induced with 3% Isoflurane in O_2_ and lubricant ophthalmic ointment (Refresh Lacri‐Lube, Allergan Ltd, Marlow, UK) was applied. Depth of anaesthesia was monitored by breathing rate and absence of pinch‐withdrawal reflex and body temperature was maintained near 37°C via a heating blanket.

#### Preparation for recordings in anaesthetized animals

Recordings were obtained from 21 animals. Surgical anaesthesia was provided by an ip injection of a mixture of 80 mg kg^−1^ ketamine and 6 mg kg^−1^ xylazine. Subsequent anaesthesia was provided by an initial ip injection of 0.1‐0.2 mL of 10% w/v urethane in 0.9% NaCl, with an additional 0.05–0.15 mL as needed. Our measurements were always obtained at least 1 h after the ketamine injection, and usually more than 1.5 h, and so we expect minimal contribution of ketamine during our measurement of visual responses (Green *et al*. 1981; Kawai *et al*. 2011; Jaber *et al*. 2014). A craniotomy was made over one hemisphere and the brain protected by agarose (2% in 0.9% NaCl). Recordings proceeded as described below for ‘acute recordings’, for 4–6 h. During the recordings, the eyes were protected by regular application of silicone oil. At the end of the experiment, animals were killed by overdose of sodium pentobarbital ip (Pentoject; Animalcare Ltd, York, UK).

#### Preparation for recordings in awake animals

Preoperative analgesia was given sc (5 mg kg^−1^; Carprieve, Norbrook, Newry, UK) and surgical anaesthesia was maintained with 1–1.5% isoflurane in O_2._. A craniotomy (8–10 mm^2^) was made in one hemisphere, centred 3.5–3.7 mm posterior to bregma, 0.7–1.1 mm lateral to the midline suture. A ground screw was implanted in the hemisphere opposite to the craniotomy, and a metal head post fixed to the skull with dental cement (Super‐Bond C&B; Sun Medical, Shiga, Japan). In six animals (acute recordings), the brain was covered with a layer of Kwik‐Cast Sealant (WPI, Sarasota, FL, USA), which was replaced with artificial cerebrospinal fluid (Bio‐Techne Ltd, Abingdon, UK) during recording sessions. In two animals (chronic recordings), the dura mater was instead removed and a custom built 16‐channel microdrive (Axona Ltd, St Albans, UK) was implanted so that electrodes were at a depth of ∼700 μm. Post‐surgical analgesic treatment was provided orally for 3 days (1 mg kg^−1^; Metacam; Boehringer Ingelheim, Ingelheim am Rhein, Germany). Animals recovered from surgery for at least 1 week and were then habituated to head‐restraint (one session per day, 8–12 sessions, 5 min on the first day and progressively increased). Two animals were supplied with a treadmill to manipulate; other animals rested on a comfortably small semi‐circular tube. The typical duration of a recording session was 90–120 min. At the end of the experiments, animals were killed by an ip overdose of sodium pentobarbital.

#### Acute recordings

Quartz/platinum‐tungsten single electrodes (impedance 4–5 MΩ) or tetrodes (impedance 0.5‐0.8 MΩ) were inserted vertically using a Mini‐Matrix system (Thomas Recordings, Giessen, Germany). The analogue signal was amplified and filtered (0.3–10 kHz), digitized and acquired at 44 kHz using the same programme that generated visual stimuli. The electrode was advanced until the surface of the SC (always located near a depth of 1.3 mm) was identified by auditory monitoring of multi‐unit (‘hash’) response to 4 Hz flicker of a large uniform field. In some animals, after the last recording, the electrode was replaced with one coated with Vybrant® DiI cell‐labelling solution (Invitrogen, Eugene, OR, USA).

#### Chronic recordings

Four independently movable tetrodes, each formed of four 12.5 μm diameter tungsten wires (impedance 0.15–0.6 MΩ), were implanted. The analogue signal was amplified and filtered (0.36–7 kHz), digitized and acquired at 48 kHz (dacqUSB; Axona Ltd). Tetrodes were lowered over several days until we functionally identified the surface of the SC as described above. Recordings were made at depth increments of ∼65 μm until robust visual responses could no longer be detected.

#### Histology

Animals were transcardially perfused with 0.1 m PBS followed by 4% paraformaldehyde in PBS, post‐fixed for 24 h in the same, and then left for 48 h in 15%, then 30% w/v sucrose in PBS. Coronal slices (CM1850 UV; Leica Microsystems, Wetzlar, Germany), 30–50 μm thick, were stained for Nissl substance or 4′,6‐diamidino‐2‐phenylindole. The histology confirmed the location of recording sites in the SC, although we do not have precise estimates of the depth of individual recording sites because we either made multiple electrode penetrations or used chronically implanted electrodes. For most recording sites, we noted the depth at which we encountered audible ‘hash’ (above) and almost all recordings were made within 500 μm of the hash (mean ± SD depth 210.4 ± 178.8 μm, *n* = 303 in awake animals).

#### Eye movements

Matlab (Mathworks, Natick, MA, USA) was used to acquire images from an infrared camera (30 Hz; DMK 22BUC03, ImagingSource, Charlotte, NC, USA) focused on the contralateral eye through a zoom lens (M3514‐MP, Computar, Cary, NC, USA) and an adjustable mirror positioned below the animal. Eye position was estimated on‐line by tracking the pupil as the centre of a fitted ellipse. We removed blinks by identifying any points where the area of the fitted ellipse was more than 3 SDs above the median. The removed values were replaced using nearest neighbour interpolation. Angular estimates of azimuth (θ_x_) and elevation (θ_y_) of the eye were calculated as:

(1) θx=180π× arcsin dx×C res r

(2) θy=180π× arcsin dy×C res r

where *r* is the radius of the mouse eye (1.25 mm; Sakatani & Isa, 2004), *C*_res_ is the camera resolution in mm pixel^−1^ at the imaging plane, and *dx* and *dy* reflect the difference between the tracked pupil centre and the average pupil centre across the measurement set. In each session, we optimized pupil imaging under the constraints of the monitor position and were not always able to track the corneal reflectance, and so we have not used a corneal reflectance as a reference point for pupil position. In six sessions with a trackable corneal reflectance, the SD of its centroid was on average 1.71° (estimated from camera pixels as above), with a range of 1.03–2.12°. The pupil was not always aligned with the axis of the camera, and so changes in pupil size can confound estimates of the pupil position. We used linear regression to factor out changes in pupil position that were probably a result of changes in pupil area.

#### Spike sorting

All recordings obtained at one site on 1 day were concatenated and analysed together. Putative single‐units were identified off‐line using Plexon Offline Sorter, version 3.3.2 (Plexon Inc., Dallas, TX, USA) for single electrode recordings or KlustaSuite (Rossant *et al*. 2016). Single‐units were identified by clustering in principal component space, followed by manual inspection of spike shape, auto‐ and cross‐correlograms. In no putative single unit did the fraction of interspike intervals under 0.5 ms exceed 2%. In 14 of 227 of visually responsive neurons in awake animals and 21 of 97 in anaesthetized animals, the fraction of interspike intervals under 1 ms moderately exceeded 2%. We have retained these units in the reported analyses; removing them did not change the conclusions.

### Visual stimuli

**Figure 1:**
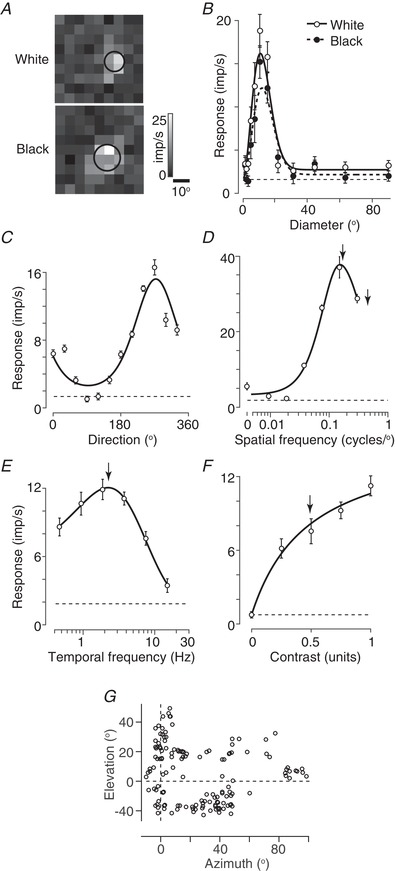
Visual responses of a representative unit recorded in superficial layers of the superior colliculus in awake mouse Each graph shows the mean response of the unit to one of the tested dimensions, and the best‐fitting prediction of the relevant model. All responses were obtained from a single unit in a single session (4‐160705‐6). The error bars are ±1 SEM over trials and dashed horizontal lines show the mean spontaneous activity measured from interleaved presentations of a grey screen. *A*, receptive field maps for flashed white or black squares (‘sparse noise’). Black circle shows 1 SD of best‐fitting Gaussian function. *B*, size‐tuning for flashed white (open symbols) or black (closed symbols) discs. The lines show the best‐fitting difference‐of‐Gaussians function in each case (solid: white; dashed: black). *C*, direction tuning for drifting sinusoidal gratings. Line shows the best fitting modified von Mises function. *D*, spatial frequency tuning (at 3.8 Hz). Line shows the best fitting difference‐of‐Gaussians function. Arrows show preferred frequency (left) and the spatial resolution (*fc*, right). *E*, temporal frequency tuning (at 0.04 cycles degree^−1^). Line shows the best fitting difference‐of‐exponentials function. Arrow shows preferred temporal frequency. *F*, contrast response function. Line the shows best fitting modified Naka‐Rushton function. Arrow shows contrast at half the fitted maximum response (*C*_50_). *G*, visual field locations of units recorded from awake animals (*n* = 144). Azimuth of 0° represents directly in front of the animal; elevation is relative to eye height.

Visual stimuli were generated using Expo (P. Lennie, Rochester, NY, USA) on a Macintosh computer (Apple Corp., Cupertino, CA, USA) and presented on a LCD monitor (awake recordings: ProLite EE1890SD; Iiyama, Hoofddorp, The Netherlands; mean luminance 35–45 candela m^−2^; anaesthetized recordings: VE228, Asus, Taipei, Taiwan; mean luminance 30–40 candela m^−2^) refreshed at 60 Hz and displaying a mean grey screen, positioned 20 cm from the animals’ eye. The monitor was gamma‐corrected by measuring the luminance of the red, green and blue elements with a photometer (Chroma meter CS‐100A; Konica Minolta, Tokyo, Japan). Neural and video recordings were aligned to the visual stimulus by recording the output of a photodiode that monitored a small corner of the stimulus monitor shielded from the animal. The coarse location of receptive fields was manually identified and the monitor location adjusted to centre them on the monitor using a flexible arm. Receptive field position estimates were subsequently refined by online analysis of responses to ‘sparse noise’, as described below. Unless specified, stimuli lasted for 2 s with an interstimulus interval of 0.5 s and were presented at the maximal contrast. Each set of stimuli included a blank condition (during which the screen was held at the mean luminance) from which ‘spontaneous’ firing rates were estimated. Each set of stimuli was presented in pseudo‐randomized order for three to 10 repetitions. Figure 1 shows the responses of an example unit recorded in an awake animal to the sets of stimuli that we primarily address.

#### Sparse noise

Uniform black or white squares were flashed for 0.2 s (no interstimulus interval) in pseudorandom sequence over a 9 × 9 grid (Fig. 1 *A*). We used either 10° squares with 5° spacing (anaesthetized animals) or 15° squares with 7.5° spacing (awake animals). For a subset of units recorded in awake animals, we compared receptive field estimates using both stimuli, and obtained similar results (not shown).

#### Size tuning

Uniform black or white circular patches (diameter 2° to 90°) were flashed for 0.5 s with an interstimulus interval of 0.5 s (Fig. 1 *B*). In some units, we also measured size‐tuning curves for a uniform field that was modulated in time by a sinusoidal waveform.

#### Drifting gratings

Circular patches of drifting sinusoidal grating (diameter 80°) varying in orientation/direction (Fig. 1 *C*), spatial frequency (Fig. 1 *D*), temporal frequency (Fig. 1 *E*) or contrast (Fig. 1 *F*). The temporal frequency was 2–5 Hz, and the spatial frequency near 0.05 cycles degree^−1^, unless varied. In some units, a large grating strongly reduced the activity such that the response was not measurable, and so we made the stimulus the largest size in which a clear response could be obtained. We measured contrast sensitivity using gratings of five or seven contrast levels; direction and orientation selectivity using gratings of 12 different directions (30° steps); spatial frequency tuning using gratings of seven or more spatial frequencies; temporal frequency tuning using gratings of six or more temporal frequencies; and speed tuning using a matrix of seven spatial frequencies and six temporal frequencies.

#### Counterphase gratings

Large circular patches (diameter 80°) of contrast‐reversing sinusoidal gratings at each of eight spatial phases (22.5° steps), with spatial frequency and orientation near that preferred by the target units, and a temporal frequency of usually 2 Hz.

### Data analysis

#### Analysis and inclusion criteria

Offline analysis was performed in the Matlab environment. Peristimulus time histograms (PSTHs) (bin width 0.01 s) were constructed for each trial. For drifting or counterphase gratings, we subjected each trial to Fourier analysis and extracted the mean firing rate (F0), the modulation amplitude and phase at the stimulus temporal frequency (first harmonic; F1) and the same for the second harmonic (F2). Unless stated, we characterize responses as stimulus‐evoked activity, which is the change in activity from that measured during presentation of a blank screen (the ‘spontaneous’ activity). Unless stated, we averaged response across the entire stimulus duration. We included in our analyses those units where this evoked response (or, if using gratings, the higher of the evoked F0 and F1 response) was at least 1.4 spk s^−1^, and was also at least 1.5 SD above the spontaneous activity (awake recordings) or 1.25 SD above it (anaesthetized recordings). The particular thresholds were chosen after manual observation of the data to define a liberal criterion for units that were probably responsive and should therefore be considered for further analysis. We further required that the centre of a unit's receptive field (estimated from the sparse noise stimulus) was within 30° of the stimulus centre (for measurements using large stimuli) or 10° of the stimulus centre (for size‐tuning curves).

For each of the various models described below, we found the set of parameters that maximized the log‐likelihood (*LL*) of the model given the responses (El‐Shamayleh & Movshon, 2011) using the Matlab function *fmincon*. In each case, we compared the model *LL* to an upper bound (*LLu*; obtained by fitting the responses to themselves) and a lower bound (*LLl;* obtained by fitting the responses to the average response across all stimuli). The normalized log‐likelihood [*LLn* = (*LL – LLl*)/(*LLu – LLl*)] was used to decide whether to include the resulting model parameters in subsequent analyses (*LLn* ≥ 0.5). In addition to the parameters of the models described below, in each case, we included an additional parameter that allowed for a spontaneous discharge rate and included in the set of responses to be modelled the activity during presentation of a blank grey screen.

#### Response latency and sustained/transient ratio

Visual response latency was obtained from responses during the ‘sparse noise’ stimulus set, for the stimulus position eliciting the largest response. We included units where two consecutive bins in the PSTH exceeded the spontaneous rate by at least 2.5 SD of that rate and performed a linear regression from the first of those bins to the bin that contained the response peak. Latency was defined as the intersection of the regression line and the spontaneous rate (Pietersen *et al*. 2014). All fits were inspected manually: where the automated procedure clearly failed, a line was fit to the rising phase of the PSTH using manually selected data points and the latency obtained as above. To quantify how sustained or transient the response was at the preferred location, we calculated the ratio of the mean response in the 0.2 s following response onset to the peak response in the same time period (Piscopo *et al*. 2013).

#### Spatial receptive field estimates

We used either Gaussian or difference‐of‐Gaussians models to describe the spatial structure of receptive fields. For responses to the ‘sparse noise’ stimulus, we found little putative contribution of a receptive field surround and therefore found the best predictions of a circular two‐dimensional Gaussian, fit independently to the mean response to black (OFF) or white (ON) stimuli (Fig. 1 *A*). Because the stimuli were relatively short, the time window for calculating mean response was obtained by finding the position of a 0.2 s time window that maximized the variance in response across stimuli (Smith *et al*. 2005). The stimuli were relatively large, and were larger than the interstimulus spacing, and so the model response was estimated by convolving the predicted receptive fields with visual stimuli rendered at a spatial resolution of 1°.

For size‐tuning curves, the model was a two‐dimensional difference‐of‐Gaussians (Rodieck, 1965; Enroth‐Cugell & Robson, 1966), fit independently to the mean response to black or white stimuli (Fig. 1 *B*). The response of the centre (*L*_e_) to a disc of diameter *d* is proportional to the integrated volume of a Gaussian:

(3) Le(d)= Ke 2π∫0de−(x/re)2dx

where *r*_e_ is the radius (at 1 SD of the peak) and *K*_e_ is the gain. A similar expression can be formed for the larger surround (*L*_i_) and the predicted response is the difference between *L*_e_ and *L*_i_. We calculated the preferred size from the model as the smallest size reaching at least 95% of the maximal response.

For spatial frequency tuning (Fig. 1 *D*), we used the frequency domain representation (Enroth‐Cugell & Robson, 1966; Croner & Kaplan, 1995):

(4) $$R\left(s\;f\right)=K_{c}\pi {r_{c}}^{2}e^{-{\left(\pi r_{c}s\;f\right)}^{2}}-K_{s}\pi {r_{s}}^{2}e^{-{\left(\pi r_{s}s\;f\right)}^{2}}$$

where *f* is the spatial frequency, *K*_c_ and *r*_c_ are respectively gain and radius (now at 1/*e* of the peak, for consistency with previous work) of the centre, and *K*_s_ and *r*_s_ are the gain and the radius of the surround. We also calculated the spatial frequency resolution of each neuron as the characteristic frequency, *fc* = 1/π*r*_c_ (Levitt *et al*. 2001).

#### Temporal frequency tuning

We used a difference‐of‐exponentials model to characterize the temporal frequency tuning (Fig. 1 *E*) (Derrington & Lennie, 1984):

(5) $$R\left(t\;f\right)=S_{1}e^{-k_{1}t\;f}-S_{2}e^{-k_{2}t\;f}$$

where *S*_1_ and *S*_2_ are scale factors and *K*_1_ and *K*_2_ are the time constants of the mechanisms.

#### Speed tuning

We measured responses to a matrix of spatial and temporal frequencies and followed standard methods to fit a two‐dimensional function in which the unit's preferred temporal frequency could depend on the stimulus spatial frequency (Priebe *et al*. 2003; Andermann *et al*. 2011; Gale & Murphy, 2014):

(6) R(sf,tf)=A× exp −( lo g2sf− lo g2sf pref )22σ sf 2× exp −[ lo g2tf− lo g2tfp(sf)]22σ tf 2

where *R*_(*sf*,*tf*)_ is the mean response to each combination of spatial and temporal frequency, *A* is the maximum response across stimuli, *sf*_pref_ is the preferred spatial frequency, *tf*_0_ is the overall preferred temporal frequency and *tf*_p_(*sf*) is the preferred temporal frequency at each spatial frequency. The relationship between preferred temporal frequency and stimulus spatial frequency is captured by:

(7) lo g2tfp(sf)=ξ( lo g2sf− lo g2sf0)+ lo g2tf0

such that ξ, the speed tuning index, is linear with respect to log spatial frequency.

#### Direction and orientation tuning

We measured response for both motion directions for each of six grating orientations. To characterize these responses (Fig. 1 *C*), we calculated direction and orientation selectivity indices (DSI/OSI), as well as global selectivity indices (gDSI/gOSI), and measures of tuning bandwidth. The DSI is:

(8) DSI =R pref −R opp R pref +R opp

where *R*_pref_ is the response to the preferred direction θ_pref_ and *R*_opp_ is the response to the opposite direction. The OSI can be defined similarly but, in this case, *R*_pref_ is the mean of responses along the preferred and anti‐preferred directions, and *R*_opp_ is the mean of responses to the two orthogonal directions. The gDSI is the amplitude of the vector sum of responses to different directions:

(9) $$\mathrm{gDSI}=\frac{\sum R_{\theta }e^{i\theta }}{\sum R_{\theta }}$$

where *R*_θ_ is the response to a grating of direction θ. The gOSI is defined in the same way but after doubling θ. The preferred direction or orientation is the angle of the relevant vector sum. To estimate tuning bandwidth, we approximated the direction tuning curve with the sum of two von Mises functions, 180° apart, each with the same bandwidth but different amplitudes:

(10) R(θ)=U+R pref ek cos (ϑ−ϑ pref )2πI0(k)+R opp ek cos (ϑ−π−ϑ pref )2πI0(k)

where *R*_pref_ is the response to the preferred direction θ_pref_ and *R*_opp_ is the response to the opposite direction, *k* is the concentration parameter of the von Mises function and *U* allows an offset (untuned) component of the tuning curve.

#### Contrast sensitivity

We characterized the contrast‐response functions (Fig. 1 *F*) using a modified Naka‐Rushton function (Peirce, 2007):

(11) R(C)=R max ×CpC50 sp +C sp

where *R* is the neuronal response given a Michelson stimulus contrast (*C*), *R*_max_ is the saturating response amplitude, *C*_50_ is the contrast at half of the maximum response amplitude and *p* is an exponent that provides for an accelerating output non‐linearity; *s* is a scaling factor that allows the exponent to be larger in the denominator and thus allows for ‘supersaturation’. We constrained *p* to be between 1 and 4 and *s* to be between 1 and 3.

### Statistical analysis

All statistical comparisons were performed in Matlab (release R2015a). Medians, as well as the mean ± SD, are presented as indicators of the shape of the relevant distributions. Reported correlations are the Pearson's correlation coefficient, *r*, unless noted. Statistical calculations were the Wilcoxon rank sum or Wilcoxon signed rank unless noted otherwise. We do not discuss statistical significance because we did not have *a priori* hypotheses for many of our observations. However, we report *P* values, to a resolution of 0.001 because they provide a relatively intuitive indication of the overlap between relevant distributions.

## Results

We characterized the receptive fields of visually responsive units in the superficial layers of the superior colliculus (sSC; including layers SZ, SGS and SO) of awake mice. Below, we show how these neurons respond to flashed stimuli and luminance modulation, and their tuning for different dimensions of a drifting grating. We show the impact of eye movements on response properties, and how that might be mitigated. Finally, we compare the response properties in awake animals with those in anaesthetized animals.

### Response to flashed stimuli

**Figure 2:**
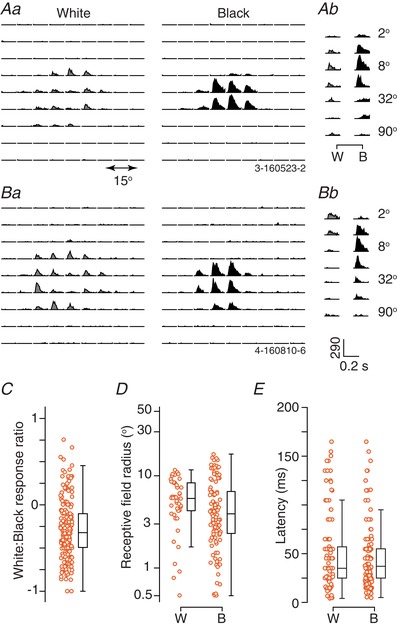
Responses to flashed white and black stimuli in awake animals *Aa*, response of a unit to white and black squares (0.2 s in duration, size 15°) presented against a grey background. The peristimulus time histogram (PSTH) at each of 81 spatial positions (separation 7.5°) is shown. *Ab*, PSTHs of the same unit as in (*Aa*), to white (W) or black (B) circular discs (0.5 s in duration) of logarithmically increasing size and centred on the receptive field. Only the first 0.2 s of the response are shown. *Ba* and *Bb*, response of another example unit. Conventions as in (*Aa*) and (*Ab*), Scale bars in (*Bb*) apply to (*A*) and (*B*). *C*, scatterplot showing the relative strength of responses to white and black stimuli, obtained from sparse noise (*n* = 219). The response ratio is *(Rw – Rb*)/(*Rw + Rb*), where *Rw* is the maximum response to a white stimulus and *Rb* is the maximum response to a black stimulus. A value of −1 (or 1) indicates a response only to black (or white) stimuli, and a value of 0 indicates an equal response to both. Here and subsequently, uniformly random offsets have been applied to the *x*‐axis to allow better visibility. The central mark in the boxplot indicates the median, and the edges indicate the 25th and 75th percentiles. The whiskers are 1.5 times the interquartile distance (99% coverage). *D*, receptive field radii (at 1 SD) obtained from best‐fitting Gaussian functions (as in Fig. 1 *A*) to white (W, *n* = 46) or black (B, *n* = 110) responses. Here and subsequently, model predictions are only shown for units where the normalized log‐likelihood of the model (see Methods) was at least 0.5. *E*, latency of visual responses from onset of white (W, *n* = 102) or black (B, *n* = 180) flashes in the preferred location, for all responsive units. [Color figure can be viewed at wileyonlinelibrary.com]

We obtained responses to flashed black or white squares presented against a grey background (‘sparse noise’) in most units. As for the examples provided in Fig. 2, many units showed brisk responses to both contrast polarities. To establish which polarity each neuron preferred, we calculated a response ratio *RR* = *(Rw – Rb)/(Rw + Rb)*, where *Rw* is the response (see Methods) to the best white stimulus and *Rb* is the response to the best black stimulus: a value of −1 (or 1) indicates a response only to black (or white) stimuli, and a value of 0 indicates an equal response to both. Most units responded better to a black stimulus than a white stimulus (174/219; 79%) and the index *RR* was a median of −0.32 (μ −0.28, SD 0.33) (Fig. 2 *C*). In many units, we were also able to measure size‐tuning curves for flashed black and white discs centred on the receptive field (Figs. 1 *B*, 2 *Ab* and *Bb*). Again, we found brisk and vigorous responses to both polarities, with the best black stimulus producing stronger responses than the best white stimulus in most units (40/46; 87%).

**Figure 3:**
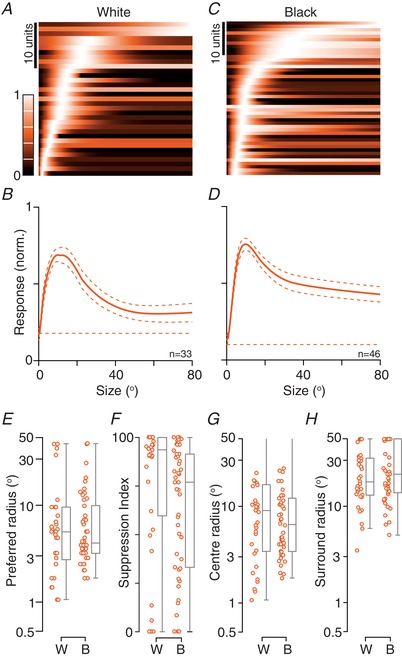
Size‐tuning for flashed white and black stimuli in awake animals *A*, population size‐tuning for a flashed white disc (duration 0.5 s) centred on the receptive field. Each row of the image shows the best‐fitting difference‐of‐Gaussians function obtained for a single unit (as in Fig. 1 *B*), normalized to its maximum response. Only units in which the normalized log‐likelihood of the model was at least 0.5 are shown. The units are ordered, from bottom‐to‐top, by the preferred size. Here and elsewhere, the colour bar applies in all cases. *B*, mean size‐tuning for a white disc, obtained by averaging across the rows in (*A*). Dashed lines show ±1 SEM. Dashed horizontal line shows the spontaneous rate, normalized to the unit's maximum visual response before averaging. *C* and *D*, same as (*A*) and (*B*) but for black discs. *E*, radius of the preferred white (W) or black (B) stimulus. *F*, percentage reduction in response from a stimulus of optimal size, to the largest tested (‘suppression index’). *G* and *H*, radius of the centre (*G*) and surround (*H*) components of the receptive field in the difference‐of‐Gaussians function. [Color figure can be viewed at wileyonlinelibrary.com]

We next considered whether the preference for black stimuli arose because receptive fields were more sensitive to black stimuli, or accumulated signals over a larger region of visual space for black stimuli. To do this, we analysed the parameters of a Gaussian model that was fit to the responses to the white or black sparse noise, including only those units where receptive fields were well described by the model. If anything, these fits showed slightly smaller receptive fields for black than white stimuli (black: median 3.9^o^, μ 5.3, SD 4.1, *n* = 110; white: median 5.1^o^, μ 5.1, SD 2.4, *n* = 35) (Fig. 2 *D*). Among those units where both polarities yielded good receptive field estimates, the centre radius was a median of 3.0^o^ for black stimuli and 5.6^o^ for white stimuli (*n* = 25; *P* = 0.093, paired Wilcoxon signed rank test). Similar analyses of a difference‐of‐Gaussians model fit to the size‐tuning curves also showed that receptive field centres were slightly smaller for black than white stimuli (Fig. 3 *E* and *G*).

Inspection of the size‐tuning curves revealed strong tuning for both white and black stimuli (Fig. 3 *A*–*D*), suggesting that the receptive field surround is sensitive to both contrast polarities. To provide an index of size‐tuning, we calculated a suppression index (SI) as the proportional reduction in response from an optimally sized to a large stimulus [SI = 100 * (*Resp*_opt_ − *Resp*_large_)/*Resp*_opt_]. This size‐tuning index was stronger for white than black stimuli (black: median SI 77.0%, μ 64.3, SD 32.7, *n* = 46; white: median 93.7, μ 76.1, SD 34.6, *n* = 33; *P* = 0.018) (Fig. 3 *F*), indicating stronger surrounds for white stimuli, whereas the receptive field surround size was similar for white and black stimuli (Fig. 3 *H*). Together, these results suggest that the preference for black stimuli in the sSC reflects greater sensitivity to black stimuli in the receptive field centre, and a greater sensitivity to white stimuli in the receptive field surround.

Stronger sensory responses are often faster responses. To establish whether neurons responded faster to black stimuli, we measured response latency during presentation of the sparse noise (see Methods) (Fig. 2 *E*). Median response latency was 35.4 ms (μ 41.9, SD 26.6, *n* = 175) for the best black stimulus, which is no different to that for the best white stimulus (median 35.0 ms, μ 50.2, SD 38.8, *n* = 98; *P* = 0.429). In 86 units in which latencies could be measured for both polarities, latencies to white and black stimuli were correlated (*r* = 0.47; *P* < 0.001), with slightly shorter responses for black than white stimuli (black: median 35.0, μ 36.2, SD 19.6; white: median 35.0, μ 45.3, SD 34.5; *P* = 0.003, paired Wilcoxon signed rank test). We conclude that sSC units prefer black over white stimuli and may respond faster to black stimuli.

We noted two types of non‐standard behaviour to flashed stimuli. First, the size tuning curves for white (but not black) stimuli in some units (9/34) was multimodal, such that response first increased with size to a peak, then declined, and then increased again (not shown). Second, although responses to black flashes in all units showed approximately circular receptive fields for sparse noise stimuli (Fig. 2 *Aa* and *Ba*), responses to white flashes were sometimes organized in a ‘donut’ shape (e.g. Fig. 2 *Ba*, left). That is, the responses to white stimuli flashed in the centre of the receptive field were less vigorous than responses to adjacent stimuli. This organization could be identified by eye in 27/98 (33.8%) units that had clear responses within the stimulus field. In the eight units where we also had size‐tuning measurements, all responded well to a small (diameter less than 8°) white disc (e.g. Fig. 2 *Bb*); and most (6/8; 75%) were strongly inhibited by large stimuli. The two other units showed the multimodal size tuning curves described above.

The responses of neurons with ‘donut’ receptive field could be well captured by supposing that an excitatory Gaussian subfield was opposed by a smaller inhibitory subfield (a difference‐of‐Gaussians model normally assumes that the inhibitory subfield, the surround, is larger than the excitatory subfield, the centre). Among neurons in which a Gaussian provided a good model of the receptive field (*LLn* > 0.5), 25/35 (71%) were better explained by the Donut model (as assessed by Akaike information criterion) (Burnham & Anderson, 2002); the Donut model could also account for a further six units in which the Gaussian model did not provide good predictions. Among these 31 units in which the Donut model provided better predictions than the Gaussian model, the inhibitory subfield was a median of 0.51 (μ 0.53, SD 0.22) of the size of the excitatory subfield and had a median radius of 3.27^o^ (μ 3.30, SD 1.53). The black receptive fields for these units were a median radius of 5.86^o^ (μ 8.07, SD 7.50) and similar in size to the excitatory white fields (median 6.41^o^, μ 6.28, SD 1.80; *P* = 0.610, paired Wilcoxon signed rank test).

### Linear and non‐linear responses to luminance modulation

**Figure 4:**
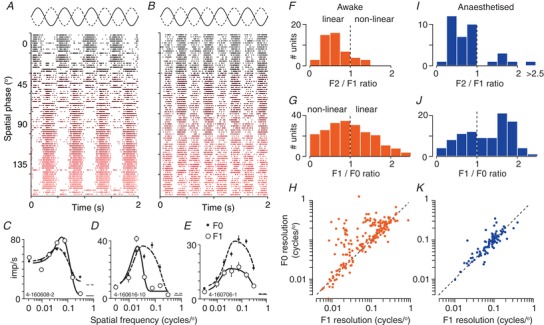
Linearity of spatial summation *A* and *B*, raster plots showing responses of a linear (*A*) and a non‐linear (*B*) unit to a stationary grating, which flickered (counterphase modulated) at 2 Hz. The schematic at top shows the temporal profile of the grating. Each row shows the spiking activity of the unit on one trial. The tick marks on the *y*‐axis separate groups of trials of the same spatial phase (also indicated by the colour of the raster). *A*, response of a unit showing linear spatial summation. Responses are modulated at the temporal frequency of stimulation. *B*, response of a non‐linear unit. Responses at all spatial phases are modulated at twice the temporal frequency of stimulation. *C–E*, amplitude of mean (F0) and modulated (F1) response as a function of the spatial frequency of a drifting grating. Horizontal lines show the F0 (dashed) and F1 (solid) for spontaneous activity. *C*, same unit as in (*A*). *D*; same unit as in (*B*). *E*, another unit. *F*, non‐linearity index (F2/F1 ratio) calculated from counterphase modulated gratings, in awake animals (*n* = 48). Values greater than 1 indicate non‐linear responses; values less than 1 indicate linear responses. *G*, linearity index (F1/F0 ratio), as calculated from drifting gratings of spatial frequency optimal for the F0, in awake animals (*n* = 227). Values larger than 1 indicate linear responses; values less than 1 indicate non‐linear responses. *H*, comparison of spatial frequency resolution for F0 and F1 responses in awake animals (*n* = 191). Values substantially above the unity line (dashed line) indicate the F0 resolves higher spatial frequencies than the F1. *I–K*, same as (*F*) to (*H*) but for units recorded in anaesthetized animals (*I*, *n* = 37; *J*, *n* = 97; *K*, *n* = 93). [Color figure can be viewed at wileyonlinelibrary.com]

If photoreceptor signals were summed linearly by a receptive field then stimuli of one polarity (e.g. white) should increase firing rate and stimuli of the other polarity (e.g. black) should reduce it. Most neurons that we encountered in the sSC, however, showed an increase in activity to both white and black stimuli, implying non‐linear operations within their receptive fields. Previous work exploring response linearity in early visual pathways has generally analysed responses to flickering (counterphase modulated) gratings. We therefore measured responses to flickering sinusoidal gratings of near‐optimal spatial frequency, presented at each of several spatial phases. A non‐linear receptive field is expected to produce responses to both the black and white phases of the stimulus (thus showing response peaks at twice the modulation frequency, or ‘F2’) and, indeed, we observed clearly non‐linear responses in a small number of units (e.g. Fig. 4 *B*). In most units, however, responses were modulated at the frequency of the stimulus (the ‘F1’) (e.g. Fig. 4 *A*) at most spatial phases. To summarize responses across the population, we followed established procedure and calculated the ratio of the mean F2 response (across all spatial phases) to the maximum F1 response (Hochstein & Shapley, 1976). The distribution of the F2/F1 ratio was unimodal, and the median ratio was 0.62 (Fig. 4 *F*). Most neurons (41/48; 85%) were classified as linear (F2/F1 ratio < 1) by this metric.

A counterpart and more widely used method for characterizing non‐linear summation is to measure the response to a drifting grating (Skottun *et al*. 1991). In this case, the activity of a linear receptive field will still be modulated at the temporal frequency of the grating (F1) but a non‐linear cell will show elevation of the mean firing rate (F0) that is only weakly modulated. We therefore calculated the F1 and F0 response to a large drifting grating (at the preferred spatial frequency for the F0 response). The median F1/F0 ratio among the units described above was 1.27, and 32/48 (67%) were classified as linear (F1/F0 ratio > 1). There was, however, only a moderate relationship between the F1/F0 ratio and the F2/F1 ratio, and we return to this below. Across the population of units for which we obtained spatial frequency tuning curves, the distribution of F1/F0 ratios was unimodal, with a median F1/F0 ratio of 0.92, and 104/227 units (46%) were classified as linear (Fig. 4 *G*). An alternative method for calculating the F1/F0 ratio would be to compare the maximum F0 response and maximum F1 response obtained across all spatial frequencies (the two measures could therefore be obtained at different spatial frequencies). As expected, this method resulted in higher F1/F0 ratios: the median F1/F0 ratio was 1.14, and 140/227 units (62%) were classified as linear (not shown).

The spatial frequency tuning of the mean (F0) and modulated (F1) response to drifting gratings distinguishes two major classes of receptive fields in early visual pathways. In some cells (eg. ‘X‐cells’ of the cat retina) (Enroth‐Cugell & Robson, 1966), the F1 response is greater than the F0 response at all spatial frequencies. In other cells (eg. ‘Y‐cells’ in cat retina), there is an F1 response at low spatial frequencies but, at higher spatial frequencies, there is an increase in F0 with little F1. Our population included units with X‐like and Y‐like spatial frequency tuning. In many neurons, the F1 response exceeded the F0 response at all spatial frequencies to which the unit responded (X‐like) (Fig. 4 *C*; same unit as in Fig. 4 *A*). Other units showed a strong F1 response at low spatial frequencies and strong F0 response at high spatial frequencies (Y‐like) (Fig. 4 *D*; same unit as in Fig. 4 *B*). Still other units showed stronger F0 response across the range of spatial frequencies to which they were responsive (Fig. 4 *E*).

The F0 response of a Y‐like unit should resolve higher spatial frequencies than the F1 response. To distinguish Y‐like units, we therefore found the best predictions of a difference‐of‐Gaussians model for spatial frequency tuning curves, fit to either the F0 or the F1 responses. Figure 4 *H* compares the spatial frequency resolution predicted by these fits (see Methods) for the 191 units in which good fits were possible for both response measures. Many units lie near the unity line, implying similar resolution for F1 and F0 response. Many units, however, lie above the unity line, showing substantially higher spatial frequency resolution for the F0 response than the F1 response (‘Y‐like’). There is, however, no clear evidence of separate functional classes. We conclude that sSC includes units with near linear responses to luminance modulation, and units with very non‐linear responses, although there are many with an intermediate profile and we see little evidence for separate functional classes.

### Influence of eye movements on responses

Similar to other animals, mice make eye movements even when their head is restrained. We aimed to determine whether these eye movements are sufficiently large to affect estimates of receptive field properties and, if they do, how we could mitigate their effects. We were particularly interested in the potential effects of eye movements on estimates of response linearity and spatial resolution. For example, imagine a linear ‘ON’ cell (i.e. responsive to white stimuli) with a receptive field ∼5° wide and presented with a grating of 0.1 cycles degree^−1^. An eye movement of 5° would shift the position of the receptive field with respect to the grating, and the result is that the receptive field would ‘see’ opposite grating phases (black, or white) in the two trials. Consequently, analyses of PSTHs that were averaged across trials would underestimate the unit's sensitivity to luminance phase. This effect would be strongest for high spatial frequencies, where relatively small eye movements can nevertheless cause large changes in stimulus phase; at low spatial frequencies, larger eye movements would be required to substantially alter stimulus (and therefore response) phase.

**Figure 5:**
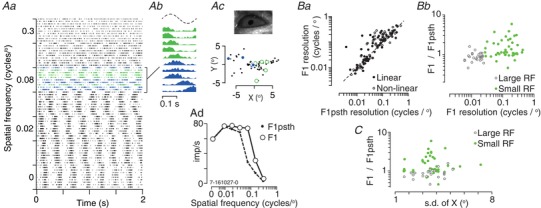
Impact of eye movements in awake animals *A*, response of a single unit to drifting gratings of varying spatial frequency (temporal frequency 4 Hz). *Aa*, each row shows the spiking activity of the unit on one trial. The tick marks on the *y*‐axis separate groups of trials of the same spatial frequency. Colours determined by average eye position in the trial (shown in *Ac*). *Ab*, cycle‐averaged PSTHs of each trial at 0.08 cycles degree^−1^, arranged by the horizontal (‘X’) eye position on that trial, with the most positive estimates at the top. The colours help indicate the horizontal eye position in each trial (shown in *Ac*). The schematic above shows one cycle of a sinusoid for comparison. *Ac*, top: image of the eye obtained during the recording session. Bottom: average pupil position on each trial, in degrees of visual angle, relative to the average of all eye positions across all trials. Larger symbols indicate trials at 0.08 cycles degree^−1^. *Ad*, spatial frequency tuning for the F1 response obtained by subjecting trial averaged PSTHs to Fourier analysis (‘F1psth’; closed symbols) or applying Fourier analysis to individual trials and averaging their amplitudes (‘F1’; open symbols). *Ba*, comparison of spatial frequency resolution for the F1 response obtained by analysing individual trials (*y*‐axis) or trial‐averaged PSTHs (*x*‐axis). Points above the line (i.e. F1/F1psth ratios greater than 1) indicate that trial averaging reduced the estimate of spatial resolution, consistent with presence of eye movements. Shown are both non‐linear (*n* = 53) and linear (*n* = 73) units, as defined by the F1/F0 ratio. *Bb*, impact of trial averaging (F1/F1psth) on estimates of spatial resolution, for linear units only. Units with small receptive fields are most affected. *C*, relationship between variability in eye position and impact of trial averaging, for linear units only. Measurements of resolution in sessions with low SD of eye position (i.e. few eye movements) are less affected by trial averaging. [Color figure can be viewed at wileyonlinelibrary.com]

Figure 5 shows that eye movements did influence our measurements of sSC responses. The raster plot in Fig. 5 *Aa* shows the spiking activity of an example unit during presentation of a vertical drifting grating of varying spatial frequency. At low spatial frequencies, the temporal profile of activity on different trials was very similar (spikes on different trials occurred at around the same time into the trial). At higher spatial frequencies, however, the activity on some trials occurred at very different times (and thus stimulus phases) compared to other trials. Figure 5 *Ab* shows PSTHs constructed from responses to a grating near the optimal spatial frequency for this unit (0.08 cycles degree^−1^). Each PSTH shows the cycle‐averaged activity of the unit for one of the trials at that spatial frequency. The trials are arranged by our estimate of average horizontal (‘X^o^’) eye position during each trial and the colours help indicate the corresponding data point in Fig. 5 *Ac*. The PSTHs peak at different phases of the stimulus cycle because the different eye positions changed the spatial phase of the stimulus relative to the receptive field. Averaging activity across the PSTHs would therefore lead us to underestimate the response modulation. To provide a straightforward measure of how inter‐trial variation in eye position influenced response, we performed Fourier analysis on PSTHs averaged across all trials (Fig. 5 *Ad*, dashed line) or performed Fourier analysis on each trial and averaged the amplitude of the F1 across trials (Fig. 5 *Ad*, continuous line) (Forte *et al*. 2002). As expected in the presence of eye movements, averaging activity across trials reduced the amplitude of the F1 at higher spatial frequencies. It should be noted that the response phase could also vary within trials and that we have not attempted to compensate for intra‐trial variation.

The responses of the example neuron in Fig. 5 *A* suggest that the impact of eye movements may be mitigated by subjecting individual trials to Fourier analysis. Because eye movements primarily affect responses to higher spatial frequencies, we expect that the benefit of trial‐by‐trial analyses will be greatest in neurons that have small receptive fields. Figures 5 *Ba* and *Bb* show that this is the case. Figure 5 *Ba* compares the spatial resolution of the F1 obtained from trial‐averaged PSTHs, as well as from individual trials, in units where adequate measurements of eye movements were available. Points above the line indicate units where trial‐averaging reduced the spatial resolution of the F1 response. As long as the F1 response was characterizable, trial‐averaging reduced spatial resolution in both ‘linear’ (F1/F0 > 1) and non‐linear units, although we restricted the following analyses to 73 linear units with well‐defined F1 responses. The impact of trial‐averaging can be summarized by calculating the change in spatial resolution brought about by trial‐averaging (ratios greater than 1 indicate that trial‐averaging reduced spatial resolution). Figure 5 *Bb* shows that the impact of trial averaging was strongest in units where spatial resolution was more than ∼0.025 cycles degree^−1^ (i.e. where the receptive field centre was smaller than about 12°). In these units, trial spatial resolution without trial averaging was a median of 1.13 times that obtained after trial averaging (geometric μ 1.54, geometric SD 2.00, *n* = 45). Units with lower spatial frequency resolution (i.e. larger receptive fields) were less affected (median 0.87, geometric μ 0.86, geometric SD 1.27, *n* = 28; *P* < 0.001).

Although our estimates of eye‐position are subject to considerable uncertainty, we were interested in determining whether they could predict the impact of trial‐averaging on spatial frequency tuning. Figure 5 *C* shows the relationship between variance in our estimates of horizontal eye position across the recording session, and the impact of trial‐averaging on spatial resolution. As expected, when the eye was relatively stable in a recording session (i.e. there was low variance), we saw little impact of trial averaging on spatial resolution.

Eye movements perturb estimates of modulated activity and may therefore also interfere with attempts to classify units as linear or non‐linear. Responses to counterphase gratings (Figs. 4 *A* and *B*) should be affected by eye movements because the temporal phase of the stimulus varies across space, and eye movements can therefore change the stimulus’ temporal phase (black, white) with respect to the receptive field. Eye movements, however, can only change the stimulus temporal phase if they cause the grating to cross the null spatial phase of the receptive field. Indeed, inspection of Fig. 4 *A* shows trial‐to‐trial variability in the response phase is limited to gratings near the unit's null spatial phase (∼45°). Consistently, when we analysed PSTHs collapsed across trials (not shown), we saw stronger ‘F2’ responses than when we analysed trial‐by‐trial activity. Trial averaged data classified 35/48 (73%) of units as ‘linear’, whereas our trial‐by‐trial analyses, reported above, yielded 85% linear units. Similarly, responses to drifting gratings should also be affected by eye movements and our estimate of F1/F0 ratio from responses to drifting gratings was lower when we used trial‐averaged F1 response: 73/227 (32%) of units would have been classified as ‘linear’ if we had used trial‐averaged responses (not shown; down from 46% linear units). For consistency across units and because the response metric is less affected by eye movements, below we use the F0 response to characterize response properties for all units.

### Contrast response functions

**Figure 6:**
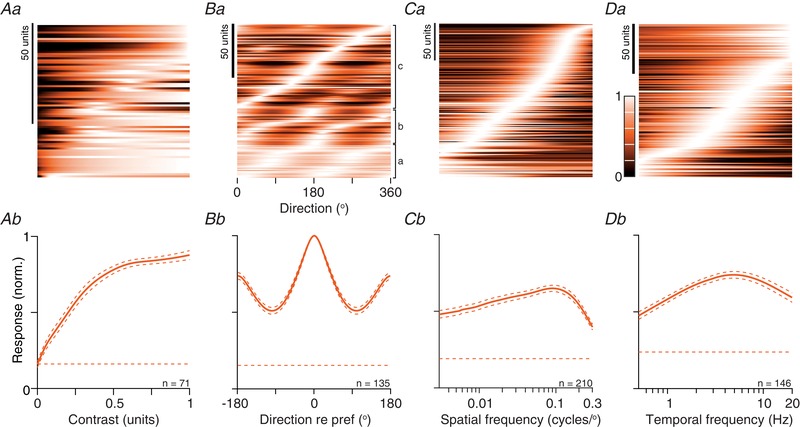
Population responses to drifting gratings in awake animals *Aa*, contrast response functions. Each row shows the predictions of a modified Naka‐Rushton function for a single unit. The units are ordered, from bottom‐to‐top, by the contrast at half‐maximal response (semi‐saturation constant, *C*_50_). Conventions as in Fig. 3 *A*. *Ab*, mean contrast response, obtained by averaging across the rows in (*Aa*). Conventions as in Fig. 3 *B*. *Ba*, orientation/direction‐tuning functions. Each row shows the predictions of a modified von Mises function for a single unit. The units are ordered by their preferred direction within each of three subgroups: neither direction, nor orientation selective (a; gOSI and gDSI < 0.1), orientation but not direction selective (b; gOSI > 0.1 and gDSI < 0.1) and remaining units (c; gDSI > 0.1). *Bb*, mean direction tuning, after aligning responses to the preferred direction. *C–D*, spatial and temporal frequency tuning. Each row in (*Ca*), shows the predictions of a difference‐of‐Gaussians function for a single unit and each row in (*Cb*), shows the predictions of a difference‐of‐exponentials function. The units are ordered by their preferred spatial or temporal frequency. *Cb*, mean spatial frequency response, obtained by averaging across the rows in (*Ca*). *Db*, mean temporal frequency response, obtained by averaging across the rows in (*Da*). [Color figure can be viewed at wileyonlinelibrary.com]

Vision is limited by the contrast sensitivity of neurons early in the visual pathway. To establish contrast response of sSC neurons, we presented a drifting grating of near‐optimal spatial frequency. We found a wide range of contrast response curves (Fig. 6 *Aa*). In some units, the response increased approximately linearly with increasing contrast. In many units, the response reached an asymptote (‘saturated’) at intermediate contrasts and further increases in contrast did not increase the response. In yet other units, the response peaked at an intermediate contrast and declined at the highest contrast (‘super‐saturating’). These various response shapes can be explained by assuming that the response of the receptive field is subject to a form of contrast gain control (Shapley & Victor, 1979) or normalization (Heeger, 1992) and that this gain control is stronger in some neurons than others. When the gain control is weak, the contrast response is approximately linear; stronger gain controls produce saturating contrast response functions, and can sometimes lead to super‐saturation (Peirce, 2007).

**Figure 7:**
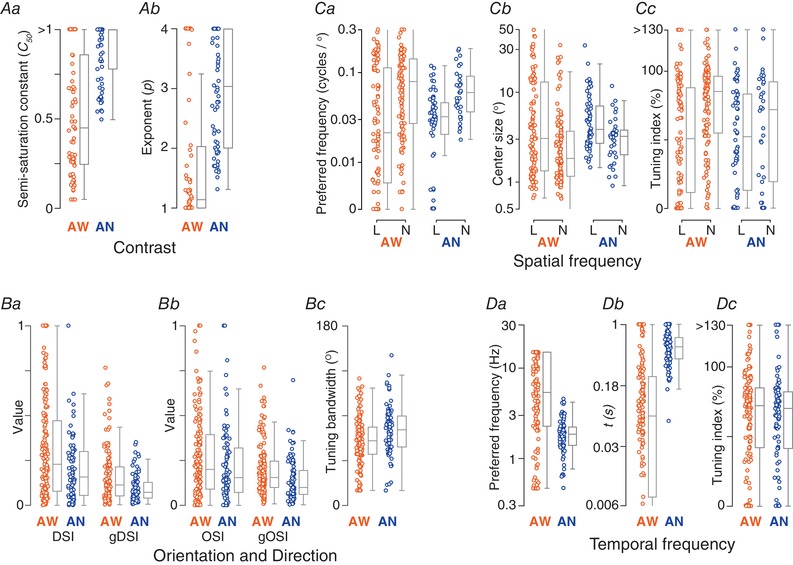
Parametric descriptions of responses to drifting gratings in awake and anaesthetized animals Units recorded in awake animals (AW) are shown to the left in orange, and those for anaesthetized animals (AN) are shown to the right in blue. *A*, parameters estimated from contrast response function fits to the tuning curves. *Aa*, semi‐saturation constant (*C*_50_, AW: 71 units; AN: 70 units). *Ab*, exponent for the expansive non‐linearity in the same units. *B*, orientation/direction‐tuning functions. *Ba*, direction‐selectivity (DSI) and global direction selectivity indices (gDSI) (AW: 151 units; AN: 86 units). *Bb*, orientation‐selectivity (OSI) and global orientation selectivity (gOSI) indices for the same units. *Bc*, bandwidth of a modified von Mises function fit to the tuning curves (AW: 135 units; AN: 81 units). *C*, parameters estimated from spatial frequency tuning fits (AW: 210 units; AN: 98 units) for linear (L) and non‐linear (N) units. *Ca*, preferred spatial frequency. *Cb*, radius of the receptive field centre. *Cc*, percentage reduction in response from preferred spatial frequency to modulation of a uniform field. *D*, parameters estimated from temporal frequency tuning fits (AW: 146 units; AN: 95 units). *Da*, preferred temporal frequency. *Db*, excitatory time constant (s). *Dc*, percentage reduction in response from preferred temporal frequency to 0.5 Hz. [Color figure can be viewed at wileyonlinelibrary.com]

To characterize responses across the population, we found the best predictions of a modified Naka‐Rushton function (Peirce, 2007) (see Methods). Across the population of units, the contrast at half‐maximum response (*C*_50_), generally used to characterize contrast sensitivity, was a median of 0.45 (μ 0.52, SD 0.33, *n* = 71) (Fig. 7 *Aa*). The exponent, *P*, that describes the initial, expansive non‐linearity was a median of 1.14 (μ 1.79, SD 1.17) (Fig. 7 *Ab*). Among the 14 units where we saw sufficiently strong super‐saturation to characterize it well (i.e. the response to the highest contrast was less than 60% that at the peak), the exponent *s* was a median of 1.89 (μ 2.03, SD 0.82; not shown).

A second indicator of the presence of gain controls is a contrast‐dependent reduction in the time to peak of the F1 response (‘phase advance’) (Shapley & Victor, 1978). In units where the F1 response exceeded the F0 response, we therefore compared time to F1 peak between the highest contrast tested and the lowest contrast at which the F1 response exceeded 5 imp s^−1^. By this metric, we saw phase advance in 18/28 units, with a median of 25.8 ms (μ 13.8, SD 8.3, *n* = 28; not shown).

### Selectivity for motion direction and grating orientation

Many neurons in the sSC of the mouse are tuned for the orientation and/or motion direction of a grating (Wang *et al*. 2010; Gale & Murphy, 2014; Inayat *et al*. 2015; Shi *et al*. 2017). For comparison with previous work, we therefore characterized direction and orientation selectivity using drifting sinusoidal gratings, usually at a spatial frequency of 0.05 cycles degree^−1^. We found a wide range of tuning for orientation and direction (Fig. 6 *Ba*). Some neurons responded well to all directions, others preferred gratings of a particular orientation but were not selective for the direction of drift, and others were selective for stimuli moving in a particular direction. Figure 6 *Bb* shows the average tuning curve across the population of neurons, after aligning each of the tuning curves to their preferred direction.

To quantify the direction and orientation selectivity of individual neurons, we calculated both widely used selectivity indices (DSI, direction; OSI, orientation), as well as global estimates of tuning (based on the circular variance, respectively gDSI and gOSI) (Fig. 7 *B*). The global selectivity indices are better overall descriptors of selectivity (Mazurek *et al*. 2014), although several studies report DSI and OSI (Niell & Stryker, 2008; Wang *et al*. 2010; Andermann *et al*. 2011; Inayat *et al*. 2015). We also fit to the data a model based on the von Mises function (see Methods), from which we extracted the bandwidth of the tuning curve (Fig. 7 *Bc*, usable fits in 135/151 units). Across the population of 151 units, 35 (23%) had a DSI greater than 0.5 (i.e. a 3:1 ratio of the response to the preferred direction over its opposite) and 25 (17%) showed a gDSI greater than 0.25, indicating strong direction selectivity. Among the latter, bandwidth was a median 59.2^o^ (μ 54.5, SD 25.9, *n* = 23). Similarly, 25/151 cells (17%) showed an OSI greater than 0.5, and 36 (24%) showed a gOSI greater than 0.25. Among the latter, bandwidth was a median 34.5 (μ 33.9, SD 13.6, *n* = 36). In agreement with previous work (Wang *et al*. 2010), we saw no bias for a particular orientation or motion direction (Fig. 6 *Ba*).

### Spatiotemporal frequency tuning

We measured responses to gratings of varying spatial or temporal frequency in most units. Above, we showed how the F0 and F1 response could be differently tuned for spatial frequency (we did not expect or see clear differences for temporal frequency tuning); the intention of this section is to provide a summary of the tuning curves and we therefore used the F0 response for all units. We used a difference‐of‐Gaussians model to capture the spatial frequency tuning of each unit (the model yielded usable fits in 210 of the 227 units). The population encompasses units with a wide range of preferred spatial frequencies, including units with low‐pass tuning, and units that responded only to the highest spatial frequencies tested (Fig. 6 *Ca*). Together, these units provided relatively uniform coverage of spatial frequencies up to about 0.2 cycles degree^−1^ (Fig. 6 *Cb*). To characterize individual units, we used the model to estimate the size of the receptive field centre (Fig. 7 *Cb*). From the fitted curves, we also obtained the preferred spatial frequency (median 0.052, μ 0.079, SD 0.078) (Fig. 7 *Ca*) and characterized the strength of tuning as the attenuation of response at low spatial frequencies (Fig. 7 *Cc*). The median receptive field centre radius was 2.34° (μ 6.90, SD 12.09), which implies a median spatial frequency resolution of ∼0.14 cycles degree^−1^ (see Methods) and 17% of units resolved at least 0.3 cycles degree^−1^ (the highest that we routinely measured). Units preferring very low spatial frequencies were more likely to be linear, and non‐linear units preferred higher spatial frequencies (*P* < 0.001). Accordingly, the receptive field centre of non‐linear units was smaller (*P* = 0.003) and the tuning curves more bandpass than linear units (*P* < 0.001).

Similar representations of population temporal frequency tuning are shown in Fig. 6 *D*. As for spatial frequency, individual units showed a wide range of tuning curves, and together provide fairly uniform coverage of temporal frequencies up to 15 Hz (the highest tested). To characterize tuning curves, we used a difference‐of‐exponentials model (Derrington & Lennie, 1984) (146/163 units yielded usable fits). From these fits, we estimated the preferred temporal frequency (Fig. 7 *Da*), the excitatory time constant (indicating the temporal frequency resolution) (Fig. 7 *Db*) and the roll off at low temporal frequencies (Fig. 7 *Dc*). Some units preferred very low temporal frequencies but most units preferred frequencies around 5 Hz (median 5.4, μ 7.4, SD 5.6) and were sharply tuned, such that very low temporal frequencies were much less effective at eliciting responses. We note that the bandwidth of the temporal frequency tuning response should indicate how sustained or transient responses are for flashed stimuli. We derived an index of sustained response for response to sparse noise (see Methods), which was a median 0.18 (μ 0.21, SD 0.11, *n* = 199) across the population (where 0 indicates a very transient response and 1 a very sustained response). Our index of temporal frequency tuning was inversely correlated with this estimate of the sustained response (*r* = −0.34, *P* < 0.001, *n* = 96). The correlation implies that, as expected in quasilinear systems, transient responses to flashed stimuli are associated with bandpass temporal frequency tuning curves, and sustained responses to flashed stimuli are associated with low‐pass temporal frequency tuning curves.

**Figure 8:**
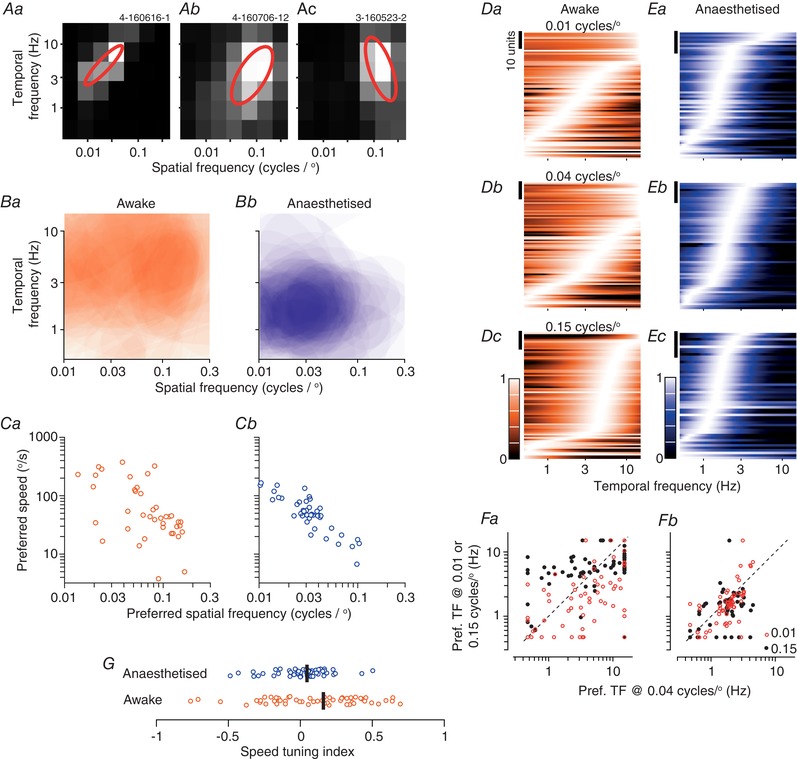
Tuning for visual speed *A*, three representative units from awake animals showing responses to a matrix of spatial and temporal frequencies. Red ellipses indicate the best fitting models described in the text. *Ba*, average spatio‐temporal tuning for units from awake animals (*n* = 56). Ellipses obtained as in (*A*) are semi‐transparent and overlaid. *Bb*, same as (*Ba*) but for units from anaesthetized animals (*n* = 62). *Ca*, relationship between preferred speed and preferred spatial frequency in awake animals. *Cb*, same as (*Ca*) but for units from anaesthetized animals. *Da*, population temporal frequency tuning functions in awake animals, for drifting gratings of low spatial frequency (0.01 cycles degree^−1^; *n* = 80). *Db*, same but for mid‐range spatial frequencies (0.04 cycles degree^−1^; *n* = 78). *Dc*, same but for high spatial frequencies (0.15 cycles degree^−1^; *n* = 75). *E*, same as *D* but for anaesthetized animals (*n* = 60, 63 and 50, respectively). *Fa*, comparison of preferred temporal frequency at mid‐range spatial frequency with that at low (open symbols, *n* = 68) or high (filled symbols, *n* = 73) spatial frequency, for units from awake animals. *Fb*, same as (*Fa*) but for units from anaesthetized animals (*n* = 50 and 60, respectively). *G*, distribution of speed tuning index in awake (*n* = 56) and anaesthetized (*n* = 62) animals. The speed tuning index is effectively the slope of the ellipses shown in (*A*), where 0 indicates that the ellipse is parallel to the temporal frequency axis, positive values indicate ellipses similar to that in (*Aa*) (0.70) and (*Ab*) (0.45) and negative values indicate ellipses similar to that in (*Ac*) (−0.38). [Color figure can be viewed at wileyonlinelibrary.com]

Selectivity for the temporal frequency of a drifting grating is important but the speed of the pattern may be more behaviourally useful. Each bar in a low spatial frequency grating moves across the screen faster than each bar in a high spatial frequency grating, and the speed of a drifting grating is its temporal frequency divided by its spatial frequency. We therefore measured responses to drifting gratings for a matrix of spatial and temporal frequencies (Fig. 8 *A*). The preferred combination of spatial and temporal frequency specifies the preferred speed of a neuron, which was widely distributed (Fig. 8 *Ba*). Preferred speed was inversely related to preferred spatial frequency (in logarithmic co‐ordinates, *r* = −0.89, *P* < 0.001, *n* = 90) (Fig. 8 *Ca*) and increased with preferred temporal frequency (*r* = 0.61, *P* < 0.001).

Although most neurons in the visual pathway are selective for the spatial and temporal frequency of a drifting grating, only a minority of neurons are tuned to grating speed. Our central question is whether there is evidence of tuning for visual speed or whether response to drifting gratings are largely independent functions of spatial and temporal frequency. To provide a model free summary of the data, we first characterized the temporal frequency tuning across the population, at each of three spatial frequencies (0.01, 0.04 and 0.15 cycles degree^−1^) (Fig. 8 *D*). The distribution of preferred temporal frequency increases with spatial frequency, suggesting that the population as a whole is speed tuned. Comparison of the preferred temporal frequency among individual units (Fig. 8 *Fa*) also shows that increase in spatial frequency generally increased the preferred temporal frequency (points lie above the diagonal), whereas a decrease in spatial frequency reduced preferred temporal frequency (points lie below the diagonal).

In plots such as that shown in Fig. 8 *A*, the responses of a speed‐tuned unit will lie along one of the positive diagonals (each diagonal is a different speed). Our population included units with clearly speed tuned responses (Fig. 8 *Aa*) and others with less clear speed tuning (Fig. 8 *Ab*). In still other units, spatial and temporal frequency tuning appeared independent or was even negatively correlated (Fig. 8 *Ac*). We used standard methods to quantify the degree of speed tuning in individual units (see Methods) (Priebe *et al*. 2003; Andermann *et al*. 2011; Gale & Murphy, 2014). Essentially, the model fits an elliptical Gaussian to the two‐dimensional response surface. One of the parameters in this model (ξ, the speed‐tuning index) determines the tilt of this Gaussian relative to the major axes. Values of ξ near 1 indicate that preferred temporal frequency is proportional to spatial frequency, and −1 indicates that preferred temporal frequency is inversely proportional to spatial frequency; a value near 0 indicates a cell in which the preferred temporal frequency is independent of spatial frequency. The model was able to account for most of the response profiles (Fig. 8 *A*). Our standard criteria admitted 76/90 units for further analysis and of these the predicted preferred stimulus was within the range of measurements in 56 units. Among these latter units, the median ξ was 0.16 (μ 0.16, SD 0.49) (Fig. 8 *G*). Similar to that observed in visual cortical areas of anaesthetized mice (Andermann *et al*. 2011), we found a weak inverse correlation between this index of speed tuning and the unit's preferred speed [*r* = −0.48, *P* < 0.001, correlation of ξ and log(preferred speed)]. Non‐linear units, which we showed above generally prefer higher spatial frequencies, also generally showed stronger speed tuning (median ξ 0.32, μ 0.25, SD 0.53, *n* = 23) than did linear units (median ξ 0.00, μ 0.10, SD 0.46, *n* = 33; *P* = 0.054; not shown). Note that units with negative ξ are common, particularly among units that preferred low spatial frequencies and high temporal frequencies. In units with negative ξ, preferred spatial frequency decreases at higher temporal frequency. This is opposite to that expected of a speed‐tuned receptive field and resembles the impact of including a surround delay into models of centre‐surround receptive fields (Enroth‐Cugell *et al*. 1983).

### Functional subclasses of receptive fields

We considered whether subgroups of units could be defined by the response properties that we measured. We collated analyses that yielded large numbers of units and relatively distinct receptive field properties, providing 10 dimensions of response variation: the black–white response ratio, response latency and index of sustained response from the sparse noise measurements, the centre size, excitatory time constant and tuning indices for spatial and temporal frequency measurements, the F1/F0 ratio, and the global direction‐ and orientation‐selectivity indices (gDSI, gOSI). Missing values were set to the mean of the relevant dimension. We used fuzzy *k*‐means clustering (Matlab Central *fuzme*)(Piscopo *et al*. 2013) to identify potential clusters in this space using the Euclidean distance between points. The number of clusters that the algorithm identifies is arbitrary. We first describe the results of this analysis when we chose five, which is our compromise between lumping and splitting. Each group included units from five to seven individual animals, except Group A (three animals).

**Figure 9:**
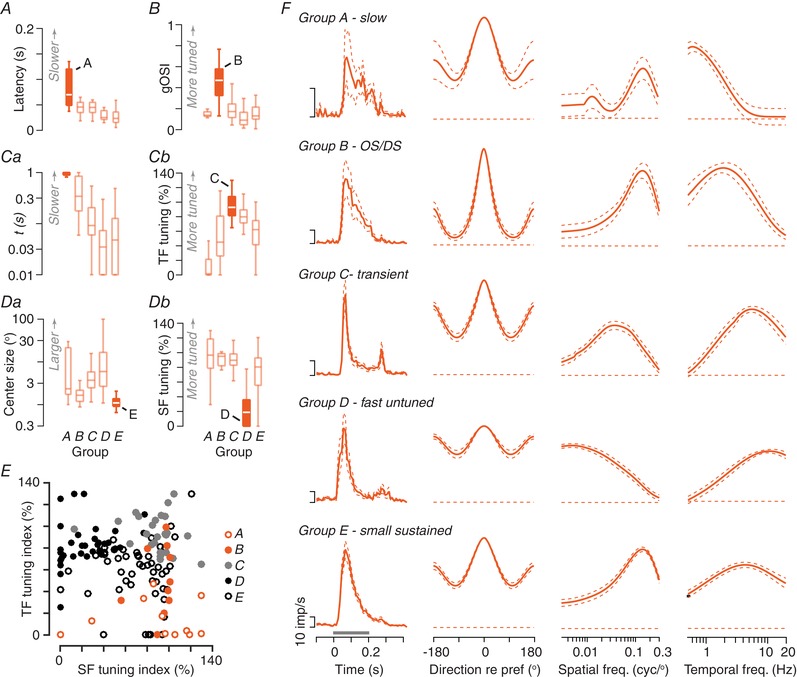
Functional subclasses of units in the sSC of awake mouse The boxplots show the tuning of five functional groupings (Groups A–E, plotted from left to right) that were identified by fuzzy *k*‐means clustering performed on measurements from 227 sSC units along 10 dimensions of response variation (see Results). The number of observations in each boxplot can vary. The white–black response ratio, index of sustained response, F1/F0 ratio and global direction selectivity indices were omitted to improve clarity. The letters and filled boxplots in some plots indicate dimension(s) of response that most distinguished each group. *A*, response latency for flashed black stimuli. *B*, global orientation selectivity index (gOSI) for drifting gratings. *C*, excitatory time constant (*Ca*) and temporal frequency tuning index (*Cb*) for the response to drifting gratings. *D*, receptive field centre size (*Da*) and spatial frequency tuning index (*Db*) for drifting gratings. *E*, comparison of spatial (as in *Db*) and temporal (as in *Cb*) tuning indices. *F*, average tuning curves for each of the groups. Left–right: response to flashed black spots (0.2 s in duration); response to drifting gratings of varying orientation and motion direction after aligning responses to the preferred direction; response to drifting gratings of varying spatial frequency; response to drifting gratings of varying temporal frequency. Response to flashed spots was simply averaged across units. Other responses were normalized to the unit's maximum response before averaging. A horizontal dashed line indicates the relative level of spontaneous activity. Other dashed lines show ±1 SEM across units. [Color figure can be viewed at wileyonlinelibrary.com]

We found that each of the five groups could be distinguished by variation along one or two dimensions in the response space (Fig. 9). Group A (13/227; 5.7%) all preferred very slowly moving gratings, with low‐pass temporal frequency tuning, low temporal resolution and long latencies (Fig. 9 *A* and *Ca*); most were linear and tightly tuned for spatial frequency. Group B (18/227; 7.9%) showed strong selectivity for the motion direction or orientation of a drifting grating (Fig. 9 *B*). These units were generally non‐linear and tuned to high spatial frequencies; their temporal properties were more variable but they generally resolved lower temporal frequencies. Group C (64/227; 28.2%) were usually non‐linear, showed highly transient response to presentation of flashing spots and were very tightly tuned for spatial and temporal frequency (Fig. 9 *Cb*). Group D (67/227; 29.5%) showed bandpass tuning for temporal frequency but low‐pass tuning for spatial frequency (Fig. 9 *Db*) and were usually linear. Less ‘speed tuning’ was also shown compared to other units (not shown). Group E (65/227; 28.6%) showed band‐pass spatial frequency tuning and were usually non‐linear; units were distinguished by small receptive fields (Fig. 9 *Da*), more sustained responses and moderate temporal frequency tuning. As is clear from the above, some dimensions of response variation were better than others at discriminating between groups; for example, the groups can almost be discriminated simply by comparing tuning for spatial‐ and temporal frequency (Fig. 9 *E*).

Using the algorithm to identify four clusters instead of five abolished the direction‐selective cluster but preserved the differences between other clusters. The main effect of increasing the numbers of clusters to six was to create two spatially low‐pass clusters (Group D above): one with larger and more linear receptive fields, and one with smaller and less linear receptive fields. In none of these analyses did the clusters reveal a preference for particular locations in the visual field.

### Comparison of responses in awake and urethane‐anaesthetized animals

Most previous measurements of sSC activity have been made in anaesthetized animals. To provide a point of comparison with the current dataset, we conducted measurements in a separate cohort of urethane‐anaesthetized mice. The responses of the populations of units that we recorded in awake and anaesthetized animals showed substantial quantitative differences, as described below.

Most sSC units recorded in anaesthetized animals, similar to those in awake animals, responded to both black and white stimuli with an overall preference for black stimuli (sparse noise: 45/65 units, 69.2%; size‐tuning: 46/68 units, 67.7%), although we encountered relatively more units that responded only to white or black stimuli (not shown). Indeed, responsivity was generally reduced in anaesthetized animals. The median F0 response (elevation above the spontaneous activity) to a large drifting sinusoidal grating of optimal spatial frequency was in anaesthetized animals 4.90 imp s^−1^ (μ 7.26, SD 11.07; *n* = 97) and the median spontaneous rate was 0.17 (μ 1.28, SD 3.32). Both evoked and spontaneous rates were lower than that obtained for the preferred spatial frequency in awake animals (evoked: median 7.34, μ 12.58, SD 19.52, *n* = 227, *P* = 0.020; spontaneous: median 1.00, μ 5.73, SD 10.74, *P* < 0.001). Similarly, measurement of contrast responses showed that the *C*_50_ increased from a median of 0.45 in awake animals to more than 1.00 (beyond which it is unconstrained) in anaesthetized animals (*P* < 0.001) (Figs. 7 *Aa* and 11 *A*). The expansive exponent, *P*, of the Naka‐Rushton function was also higher in anaesthetized animals, implying a greater impact of threshold (Fig. 7 *Ab*) and only three units required a super‐saturation term to explain their responses (not shown).

The reduced responsivity in anaesthetized animals was particularly prominent at high temporal frequencies, while units in awake animals preferred a median of 5.4 Hz, units in anaesthetized animals preferred median of 1.8 Hz (μ 1.9, SD 0.8, *n* = 95; *P* < 0.001) (Figs. 7 *Da* and 11 *D*). Consistently, the excitatory time constant, an indicator of sensitivity to high temporal frequencies, was substantially longer in anaesthetized animals (Fig. 7 *Db*) and the tuning index was weaker (Fig. 7 *Dc*). Also, consistently, anaesthesia had profound influence on response latency: median latency was 145.0 ms (μ 137.8, SD 36.5, *n* = 32) in response to white flashes and 125.0 (μ 130.6, SD 23.4, *n* = 49; *P* = 0.026) in response to black flashes, with both being substantially longer than in awake animals (*P* < 0.001 in both cases).

**Figure 10:**
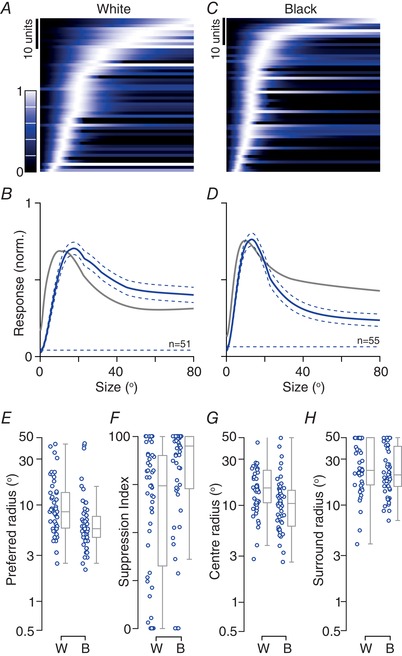
Size‐tuning for white and black stimuli in anaesthetized animals Same conventions as Fig. 3. [Color figure can be viewed at wileyonlinelibrary.com]

**Figure 11:**
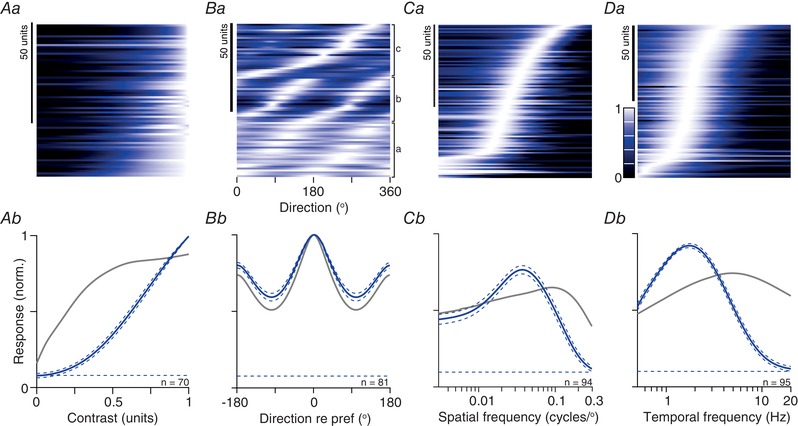
Population responses to drifting gratings in anaesthetized animals Same conventions as Fig. 6. Grey lines in (*Ab*) to (*Db*), replot the average population responses from awake animals in Fig. 6. [Color figure can be viewed at wileyonlinelibrary.com]

Although sensitivity and high temporal frequency responsivity were strongly affected by anaesthesia, the spatial profile of receptive fields appeared to be less affected. The distribution of preferred spatial frequency was similar for awake and anaesthetized animals (*P* = 0.068) (Figs. 7 *Ca* and 11 *C*), as was the index of spatial tuning for drifting gratings (*P* = 0.543) (Fig. 7 *Cc*). Anaesthesia did, however, lead to an overall increase in receptive field size estimated from gratings (from a median radius of 2.3^o^ to 3.5^o^, *P* < 0.001) (Fig. 7 *Cb*) and reduced the population coverage of high spatial frequencies (Fig. 11 *C*). Similarly, size‐tuning for uniform fields (Fig. 10) was qualitatively similar in anaesthetized and awake animals, although there were quantitative differences. First, receptive fields were larger in anaesthetized animals, whether measured with white (*P* = 0.002) (Fig. 10 *B*) or black (*P* = 0.005) (Fig. 10 *D*) spots. Second, in awake animals, spatial tuning (Fig. 3 *F*) was slightly stronger for white than black spots, whereas, in anaesthetized animals the pattern was reversed (*P* = 0.001) (Fig. 10 *F*). Finally, we saw no clear examples of ‘donut’ receptive fields in anaesthetized animals, although the Donut model provided (slightly) better predictions than the Gaussian model in 10/33 units.

The reduced response at high spatial frequencies appeared to reflect a stronger impact of anaesthesia on non‐linear units, among which receptive field centre sizes were larger in anaesthetized animals (*P* = 0.024). The populations of linear units showed less difference between awake and anaesthetized animals. Indeed, neurons had a greater probability of showing linear responses in anaesthetized animals (F1/F0 ratio > 1) (Fig. 4 *J*) and we saw little evidence of ‘Y‐like’ units in anaesthetized animals (Fig. 4 *K*). Furthermore, speed‐tuning was more pronounced among non‐linear units in awake animals, and average speed tuning was lower in anaesthetized animals (the speed index was a median 0.04, μ 0.02, SD 0.17, *n* = 62 in anaesthetized animals; *P* = 0.054) (Fig. 8 *G*).

Selectivity for grating orientation was similar in awake and anaesthetized animals (Figs. 7 *Bb* and 11 *B*). We note that sometimes, during the experiments in anaesthetized animals, a smaller grating was used to allow a robust response from the neuron under investigation. The orientation spectrum of a grating viewed through a small window is broader than that viewed through a large window. Indeed, we found a positive correlation between stimulus size and gOSI in anaesthetized animals (*r* = 0.37, *P* < 0.001). When we considered only neurons tested with gratings larger than 35° in diameter, there was no difference in orientation tuning in awake and anaesthetized animals. We did, however, encounter relatively fewer directionally selective units in anaesthetized animals (6/86; 7% showed gDSI > 0.25) even when we considered only those units tested with large gratings.

Cluster‐based analyses of receptive field properties in anaesthetized animals (not shown), analogous to those performed for awake animals in Fig. 9, produced clusters that resembled the ‘slow’ and ’OS/DS’ groups (Groups A and B) in awake animals, as well as a cluster of units with low‐pass spatial tuning similar to Group D. We did not see strongly transient units (Group C), although we note that the preferred temporal frequency of all units was much reduced during anaesthesia.

### Relationship between receptive field properties and location in the visual field

We considered whether there was obvious dependence of receptive field properties on location of the receptive field in the visual field in awake or anaesthetized animals. We found a weak inverse correlation of receptive field centre size (estimated from the spatial frequency tuning curve) and receptive field elevation (awake: *r* = −0.37, *P* < 0.001, *n* = 99; anaesthetized: *r* = −0.35, *P* < 0.001, *n* = 90), indicating that neurons in the upper visual field could detect higher spatial frequencies. Similarly, we found a weak inverse correlation between the excitatory time constant and receptive field elevation (awake: *r* = −0.33, *P* = 0.003, *n* = 79; anaesthetized: *r* = −0.25, *P* = 0.018, *n* = 91), indicating that neurons in the upper visual field could detect higher temporal frequencies. Our measures of orientation and direction selectivity did not clearly depend on receptive field elevation, and we saw little dependence of any of these measures on receptive field azimuth (*P* > 0.1 in all cases). The one exception was that there was a weak positive correlation between azimuth and receptive field centre size in awake animals; *r* = 0.28, *P* = 0.005, *n* = 99), indicating that nasal receptive fields could detect higher spatial frequencies. We note that while we recorded from a range of visual field elevations in the nasal visual field (−20° to +40°), our recordings from the temporal visual field were primarily in lower visual field, near −20° (Fig. 1 *G*). The non‐uniform sampling makes it difficult to be definitive about the relationship between elevation, azimuth and receptive field properties.

### Comparison of responses to flashed, flickering and moving stimuli

**Figure 12:**
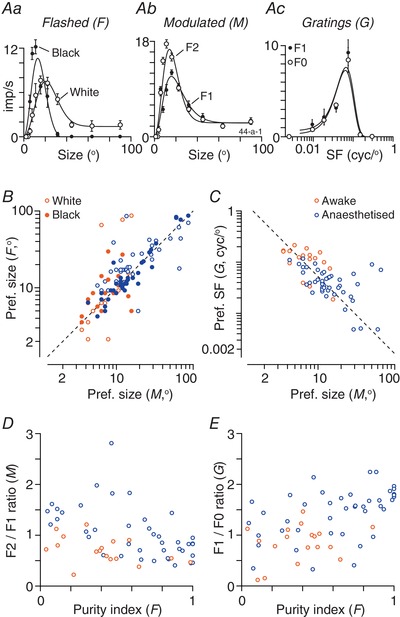
Comparison of responses to flashed and modulated stimuli *A*, response of a representative sSC unit recorded in an anaesthetized animal. *Aa*, mean response to flashed white or black discs (0.5 s in duration) of varying size. *Ab*, response to sinusoidal modulation of a uniform field of varying size, at the frequency of modulation (2 Hz; ‘F1’) or twice that frequency (‘F2’). *Ac*, mean (F0) and modulated (F1) response to large drifting gratings of varying spatial frequency (drifting at 3.8 Hz). *B*, comparison of preferred size for modulated fields (‘M’, as obtained in *Ab*) and flashed stimuli (‘F’, as obtained in *Aa*). Measurements from white (*n* = 54) and black flashes (*n* = 59) are overlaid and most units contribute to both datasets. Dashed line indicates unity line. *C*, comparison of preferred size for modulated fields (‘M’) and preferred spatial frequency for drifting gratings (‘G’, as obtained in *Ac*) (*n* = 66). Dashed line is a linear regression in logarithmic co‐ordinates. *D*, comparison of response purity for flashed stimuli, with index of non‐linearity for modulated fields (F2/F1 ratio; *n* = 59). Purity indices of 1 indicate response only to white or black, whereas 0 indicates equal response to both. Purity index and F2/F1 ratio are both derived from responses to the preferred size, obtained from modulated fields. *E*, comparison of response purity for flashed stimuli and index of linearity for drifting gratings (F1/F0 ratio, calculated at the optimal spatial frequency for the F0; *n* = 60). [Color figure can be viewed at wileyonlinelibrary.com]

We have presented responses to both flashed stimuli and drifting gratings, both of which can yield estimates of spatial receptive field properties and response linearity. We were also interested in determining whether these measurements were consistent within individual units. To explore this, we considered only units in which we were able to obtain size‐tuning curves for both flashed stimuli (Fig. 12 *Aa*) and flickering stimuli (sinusoidal modulation of a uniform field) (Fig. 12 *Ab*), as well as spatial frequency tuning curves for large patches of drifting grating (Fig. 12 *Ac*). In each case, we included only units where the best fitting difference‐of‐Gaussians function provided a normalized log likelihood of at least 0.5. To increase power, we combined measurements from awake and anaesthetized animals.

We first compared size‐tuning curves obtained for flashed white and black stimuli with those obtained with modulated stimuli. Encouragingly, preferred size was similar for modulated and flashed stimuli (Fig. 12 *B*). Preferred size for modulated stimuli (obtained from the mean rate) could be predicted either by responses to white flashes (*r* = 0.69 in logarithmic co‐ordinates, *P* < 0.001, *n* = 54) or black flashes (*r* = 0.85, *P* < 0.001, *n* = 59). The strength of size tuning for modulated stimuli, as captured by the ‘suppression index’, could also be predicted by size tuning for either white or black flashes (respectively *r* = 0.70 and 0.73 in linear co‐ordinates, both *P* < 0.001; not shown).

We next considered whether spatial receptive fields obtained with size‐tuning curves could predict those obtained with drifting gratings. Again, encouragingly, the preferred size for modulated stimuli was inversely related to the preferred spatial frequency (*r* = −0.73, *P* < 0.001, *n* = 66) (Fig. 12 *C*). That is, units that preferred low spatial frequencies also preferred large spots. Similarly, the strength of tuning revealed by size‐tuning was similar to that revealed by the spatial frequency tuning curves (*r* = 0.82, *P* < 0.001, *n* = 66; not shown). Finally, the difference‐of‐Gaussian models fit to both size‐ and spatial frequency tuning consistently organized centre and surround receptive field sizes (*r* = 0.60 and 0.45, *P* < 0.001 in both cases, *n* = 66; not shown).

We then considered whether the strength of response to flashed black and white stimuli could predict how a unit responds to luminance modulation. For example, if a unit responds only to black stimuli, and not to white stimuli, then we might expect it to show a clear F1 response to flickering spots or drifting gratings; conversely, if it responds equally well to black and white stimuli, then it should show a F2 response to flickering spots and a F0 response to drifting gratings. To test this prediction, we found the stimulus size that generated maximum response for modulated stimuli (Fig. 12 *Ab*). We then derived an index of black‐white purity index from the responses to flashed stimuli of this size [*Purity* = mod(*Rb – Rw*)/(*Rb* + *Rw*)]. Purity indices near 1 indicate a response only to black or to white stimuli; indices near 0 indicate an equal response to black and white stimuli. To compare flashed and modulated uniform fields, we compared this purity index with the F2/F1 ratio for flickering uniform fields of the same size (Fig. 12 *D*). There is substantial variability, but units with low F2/F1 ratios (i.e. linear units) usually showed high purity indices for flashed stimuli, whereas units with higher ratios (i.e. non‐linear units) generally showed lower purity indices (*r* = −0.25, *P* = 0.059, *n* = 59). The purity index could better predict the F1/F0 ratio for drifting gratings of optimal spatial frequency (Fig. 12 *E*): units with higher purity had higher F1/F0 ratios (*r* = 0.54, *P* < 0.001, *n* = 60). Together, these results suggest that non‐linear responses to luminance modulation reflect the presence of excitatory responses to both contrast polarities.

## Discussion

We have provided a quantitative analysis of the visual receptive field properties in the sSC in awake mice. We find that receptive fields are (i) usually ‘ON–OFF’ with a general preference for black stimuli; (ii) highly sensitive with brisk, short latency visual responses; (iii) weakly non‐linear in response to luminance modulation; and (iv) often speed‐tuned or direction‐selective. The constellation of receptive field properties appears to consist of at least five functional subclasses. We also show that measurements of receptive field properties in awake animals are susceptible to eye movements, as well as how they may be mitigated in analyses. Qualitatively similar responses were obtained in awake and urethane‐anaesthetized animals, although receptive fields in awake animals have higher contrast sensitivity, shorter visual latency and a stronger response to high temporal frequencies.

### Comparison with previous work

Most of our observations in anaesthetized animals are consistent with previous comprehensive functional studies (Wang *et al*. 2010; Gale & Murphy, 2014), which find similar distributions of preferred size, spatial frequency, temporal frequency, direction and orientation selectivity, although we find fewer speed‐tuned cells than Gale & Murphy (2014). The preference that we see for black stimuli was not apparent in Wang *et al*. (2010). We note, however, that we measured ‘OFF’ responses with black stimuli, whereas Wang *et al*. (2010) measured them from the offset of white stimuli. Where there is overlap, our measurements in awake animals are also consistent with a recent study by Ito *et al*. (2017) reporting similar distributions of spatial frequency preference, linearity of spatial summation, and prevalence of orientation and direction selectivity.

One potentially puzzling observation in both our work and that of Ito *et al*. (2017) is the prevalence of largely linear responses to counterphase modulated gratings, despite a general prevalence of ON–OFF responses for flashed stimuli and less linear responses to drifting gratings. A spiking threshold might hide the second‐harmonic response to counterphase modulation if the input of one (ON or OFF) subfield is substantially stronger than the other and, indeed, most sSC neurons preferred black stimuli. The fact that most units showed a null spatial phase suggests that summation with the ON and OFF subfields might be approximately linear.

We identified at least five functional groups of units in our recordings from awake animals. Anatomical studies of rodent sSC show at least four morphologically distinct cell classes (including ‘horizontal’, stellate’, ‘narrow field’ and ‘widefield’) (Langer & Lund, 1974; Gale and Murphy, 2014). In anaesthetized mice, the functional properties of the four classes are overlapping; for example, all of the morphological classes showed some orientation or direction selectivity (Gale & Murphy, 2014). Other functional properties were more distinct. Specifically, horizontal cells showed large receptive fields with little speed, orientation or direction selectivity, and a preference for low spatial frequencies; they probably constitute many of our Group D units. Widefield cells preferred the lowest speeds and may be among our Group A units, although, as in our measurements from anaesthetized animals, most morphological classes in Gale and Murphy (2014) preferred low temporal frequencies. Stellate and narrow field cells resolved the highest spatial frequencies and may be in our Group E units. Our analyses also suggest some overlap between functional subclasses in the sSC and LGN of the mouse: our Groups A and B align well with the ‘slow’ and ‘orientation and direction selective’ clusters identified in the LGN of anaesthetized mice (Piscopo *et al*. 2013). Additionally, similar to the work in the LGN, we find evidence for very transient (Group C) and more sustained (Group E) units, except that few neurons respond exclusively to ON or OFF stimuli in the sSC.

Mouse retinal ganglion cells cluster into at least 30 functional output channels with distinct properties (Baden *et al*. 2016). Most retinal ganglion cells (RGCs) project to the SC (Ellis *et al*. 2016), including ON, OFF and ON–OFF RGCs. The projections of different subtypes of RGCs (defined by their dendritic morphology) have been shown to display distinct and stereotyped axonal arborizations patterns in the SC (Hong *et al*. 2011). Surprisingly, ON projections are more probable than OFF or ON–OFF (Ellis *et al*. 2016), and so the fact we see stronger response to black stimuli suggests that the gain of OFF inputs may be selectively increased. Genetically identified subpopulations of ganglion cells that project to the SC include F‐RGCs (resembling primates’ midget RGCs, with either ON or OFF responses; some are directional), α‐RGCs, the non‐directional ON–OFF W3 RGCs, and the directional OFF J‐RGCs and ON‐OFF BD‐RGCs (Kim *et al*. 2010; Zhang *et al*. 2012; Dhande & Huberman, 2014; Rousso *et al*. 2016). W3 cells have small ON–OFF receptive fields with high spatial resolution, are non‐linear, and are sensitive to moving stimuli (Zhang *et al*. 2012). The dependence of receptive field properties on layer within the sSC is not yet clear, except that direction selectivity is more pronounced in the uppermost layers (Inayat *et al*. 2015; Ito *et al*. 2017; Shi *et al*. 2017), consistent with the projection of direction selective retinal ganglion cells (Kim *et al*. 2008; Huberman *et al*. 2008 *a*; Huberman *et al*. 2009; Kim *et al*. 2010; Hong *et al*. 2011; Kay *et al*. 2011; Dhande & Huberman, 2014; Martersteck *et al*. 2017).

The functional properties of W3 RGCs resembles that of many of the non‐linear units we encountered in the sSC (e.g. our Group E). The functional properties of retinal W3 cells resemble those of the local edge detector described in rabbit retina (Levick, 1967; van Wyk *et al*. 2006). Local edge detector units are suppressed when edges appear in the receptive field surround and units may therefore respond better to an offset white spot than a centred one. This may help explain the ‘donut’‐like receptive fields that were prominent amongst Group E units: in 19/25 (76%) of Group E units where we could characterize the receptive field, responses to white flashes were better explained by the Donut model.

### Impact of eye movements in awake animals

Our measurements show that eye movements in mice are sufficiently large to produce clear effects on the visual response, and can confound some analyses. The response of a linear receptive field is by definition dependent on the position (equivalently, phase) of any stimulus, and eye movements shift the position of stimuli with respect to receptive fields. We show that, to some degree, the impact of eye movements can be mitigated by analysing individual trials where those trials have relatively short durations. In the mouse, eye movements are relatively small and do not appear to be goal directed, and the necessary translation between retinal and head‐centred co‐ordinate frames may therefore be negligible compared to monkeys. Whether neurons in the mouse SC, similar to those in primate SC, help transform retinal to spatial co‐ordinate frames may be a question worth pursuing.

### Effect of anaesthesia

Consistent with previous work in the dLGN and V1 of the mouse (Vaiceliunaite *et al*. 2013; Durand *et al*. 2016), we found that anaesthesia (provided by urethane in the present study) induced changes in a variety of functional properties. The most prominent differences are a marked decrease in contrast sensitivity in anaesthetized animals, lower spontaneous activity and reduced responsivity, particularly to high temporal frequencies. These reductions in activity, sensitivity and responsivity are accompanied by a pronounced increase in response latency. We also observed weaker responses to high spatial frequencies in the SC in anaesthetized animals, and analyses of size‐tuning curves also suggest that receptive field sizes may be larger in anaesthetized animals. We note that units with smaller receptive fields (or at least more sensitive to high spatial frequencies) were predominantly non‐linear. Non‐linear units were also more likely to be speed tuned, and we found that speed tuning was weaker in anaesthetized than awake animals. In addition, we saw relatively more units with very linear responses (high F1/F0 ratios) (Fig. 4) in anaesthetized animals. This may reflect the increased contribution of the spiking threshold to responses under anaesthesia (see above) or a reduced response to high spatial frequencies, where non‐linear responses are more pronounced (Wang *et al*. 2010). Thus, anaesthesia may have stronger impact on the activity of non‐linear neurons, or the non‐linear components of their receptive fields.

We do not know the origin of the functional differences that we see between awake and anaesthetized animals because there are several potential mechanisms mediating the effect of anaesthesia on the spontaneous and evoked firing rate (Sceniak & Maciver, 2006; Haider *et al*. 2013; Wang *et al*. 2014). One intriguing possibility is that anaesthesia may also have strong impact on recurrent networks in the sSC that are probably important in building response properties. For example, direction tuned neurons in the sSC receive directionally tuned input from the retina that is amplified by intracollicular excitatory connections between neurons with similar directional preferences (Shi *et al*. 2017); these may be reduced by anaesthesia (Wang *et al*. 2014). Similarly, intracollicular inhibitory connections are probably important in shaping functional tuning (Inayat *et al*. 2015) and anaesthesia‐dependent changes in inhibition may be important, as in the primary visual cortex (Haider *et al*. 2013). Finally, in addition to changing the biophysical properties of SC neurons, anaesthesia probably also influences neurons sending the inputs to the SC. Reduced retinal activity cannot be ruled out. Feedback from primary visual cortex has also been shown to boost the gain of SC neurons (Zhao *et al*. 2014) and the firing rate of V1 neurons decreases under anaesthesia (Vaiceliunaite *et al*. 2013).

### Relation to other visual pathways and behaviour

Our observations show that population response in awake sSC peaks around 0.1 cycles degree^−1^ and can resolve substantially higher than 0.3 cycles degree^−1^. Retinal (pattern electroretinogram, or PERG) measurements from the anaesthetized C57BL/6J mouse show a resolution of around 0.6 cycles degree^−1^ (Porciatti, 2007). Behaviourally, mice show optomotor responses that peak near 0.1 cycles degree^−1^, and resolve around 0.5 cycles degree^−1^ (Umino *et al*. 2008) and mice can reliably detect presence of gratings of around 0.5 cycles degree^−1^ (Prusky *et al*. 2000). Similarly, our population temporal frequency tuning peaks near 6 Hz, and resolves greater than 15 Hz. Optomotor responses peak near 1 Hz and resolve more than 10 Hz, but other behavioural measures are not available. PERG and standard ERG measurements also suggest the photopic response of cone pathways peaks near 6 Hz, although this is substantially attenuated by 15 Hz (Krishna *et al*. 2002). The contribution of different post‐receptoral pathways to the ERG is not clear, but the high temporal frequency resolution that we see may be consistent with amplification of OFF‐pathway signals in the sSC because OFF‐pathway signals may be relatively important in the ERG at high temporal frequency (Tanimoto *et al*. 2015).

The superficial layers of the SC are generally considered to provide visual analyses complementary to those performed by the geniculo‐cortical visual pathway. Neurons in V1 have a somewhat stronger orientation and direction selectivity than those in the SC (Andermann *et al*. 2011; Durand *et al*. 2016), suggesting that it may have a more prominent role in analysing spatial form. Yet neurons in the SC, dLGN and V1 prefer similar spatial frequencies (Andermann *et al*. 2011; Durand *et al*. 2016) and it is difficult to infer which may be more important in fine‐grained spatial analysis. Our observations also suggest that neurons in the SC prefer higher temporal frequencies and speeds than those in the V1 (Andermann *et al*. 2011; Durand *et al*. 2016). Interestingly, although SC lesions do not influence the velocity tuning of mouse V1, they do influence the high‐speed responses of higher visual cortical areas, via projection‐specific subdivisions of the lateral posterior nucleus of the thalamus (Tohmi *et al*. 2014).

## Additional information

### Competing interests

The authors declare that they have no competing interests.

### Author contributions

GDF and SGS designed the experiments, performed the data analysis, interpreted data, wrote the paper, approved the final version of the manuscript submitted for publication, and agreed to be accountable for all aspects of the work in ensuring that questions related to the accuracy or integrity of any part of the work are appropriately investigated and resolved. GDF performed the experiments. All persons designated as authors qualify for authorship, and all those who qualify for authorship are listed. Experiments were performed at the Institute of Behavioural Neuroscience, University College London (UK).

### Funding

SGS received support from the People Programme (Marie Curie Actions) of the European Union's Seventh Framework Programme (FP7/2007‐2013) under REA grant agreement no. 618661, and a project grant from the Biotechnology and Biological Sciences Research Council (BB/R004765/1). G.D.F. was also supported by an Impact studentship from UCL.
